# Searching for biomarkers in schizophrenia and psychosis: Case‐control study using capillary electrophoresis and liquid chromatography time‐of‐flight mass spectrometry and systematic review for biofluid metabolites

**DOI:** 10.1002/npr2.12223

**Published:** 2021-12-08

**Authors:** Saehyeon Kim, Satoshi Okazaki, Ikuo Otsuka, Yutaka Shinko, Tadasu Horai, Naofumi Shimmyo, Takashi Hirata, Naruhisa Yamaki, Takaki Tanifuji, Shuken Boku, Ichiro Sora, Akitoyo Hishimoto

**Affiliations:** ^1^ Department of Psychiatry Kobe University Graduate School of Medicine Kobe Japan; ^2^ Department of Neuropsychiatry Faculty of Life Sciences Kumamoto University Kumamoto Japan; ^3^ Department of Psychiatry Yokohama City University Graduate School of Medicine Yokohama Japan

**Keywords:** biomarkers, metabolomics, psychosis, schizophrenia, systematic review

## Abstract

Metabolomics has been attracting attention in recent years as an objective method for diagnosing schizophrenia. In this study, we analyzed 378 metabolites in the serum of schizophrenia patients using capillary electrophoresis‐ and liquid chromatography‐time‐of‐flight mass spectrometry. Using multivariate analysis with the orthogonal partial least squares method, we observed significantly higher levels of alanine, glutamate, lactic acid, ornithine, and serine and significantly lower levels of urea, in patients with chronic schizophrenia compared to healthy controls. Additionally, levels of fatty acids (15:0), (17:0), and (19:1), cis‐11‐eicosenoic acid, and thyroxine were significantly higher in patients with acute psychosis than in those in remission. Moreover, we conducted a systematic review of comprehensive metabolomics studies on schizophrenia over the last 20 years and observed consistent trends of increase in some metabolites such as glutamate and glucose, and decrease in citrate in schizophrenia patients across several studies. Hence, we provide substantial evidence for metabolic biomarkers in schizophrenia patients through our metabolomics study.

## Abstract 2

In this study, we analyzed 378 metabolites in the serum of schizophrenia patients using CE and LC‐TOFMS. With a systematic review, here we provide substantial evidence for metabolic biomarkers in schizophrenia patients through our metabolomics study.

## Graphical abstract

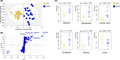

## 1 INTRODUCTION

Schizophrenia is a chronic and disabling psychiatric illness that affects around 1% of the general population.^1^ Previous studies suggest that neuronal dysfunction in dopaminergic and glutaminergic systems,^2^ metabolic abnormalities in glycolysis and oxidative stress responses, and hyperactive immune systems could be involved in the pathogenesis of schizophrenia.^3^

Neurochemical imaging tools, such as magnetic resonance spectroscopy, positron emission tomography, and magnetic resonance imaging, have long been used along with salivary cortisol and blood‐based biomarkers (inflammatory, oxidative stress, immune analytes)^4^ in the pathological study of schizophrenia. Recently, metabolic biomarkers have attracted attention. The number of studies trying to identify biomarkers of schizophrenia and other psychotic disorders in biofluids (blood, urea, cerebrospinal fluid), the post‐mortem brain, and through magnetic resonance spectroscopy has rapidly grown in recent years.^3^ According to previous systematic reviews,^3^, ^5^ glutamate, *N*‐acetyl aspartate, and some lipids and lipid‐like molecules are candidates for biomarkers that may be involved in the pathogenesis of schizophrenia. However, no robust biomarkers have been identified thus far.

Mass spectrometry (MS) coupled with gas chromatography, liquid chromatography (LC) or capillary electrophoresis (CE), and nuclear magnetic resonance (NMR) spectroscopy has been commonly used for biofluid analysis.^3^, ^6^ As there is no single analytical platform capable of detecting all metabolites, an integrated approach using more than one platform is often adopted in recent studies to provide the most sensitive and reliable measurements.^6^

Here, we analyzed 378 metabolites in the serum, including fatty acids, amino acids, and other organic acids in patients with schizophrenia and healthy controls, by combining CE‐time‐of‐flight mass spectrometry(TOFMS) and LC‐TOFMS. We subjected these data to multivariate analysis and tried to identify the metabolites that can differentiate patients with schizophrenia from healthy control subjects to build a biomarker that can assess the disease trait of patients. We also compared the acute and remission state of the same patients and tried to identify a biomarkers corresponding to the difference between these two states.

In biomarker research, univariate analysis using Mann‐Whitney or predictive models is more commonly used, and there are not so many reports of studies using multivariate analysis. Multivariate analysis has been attracting attention in recent years and is now being used in the field of metabolomics.^7^, ^8^ The metabolome is actually a result of the intersection of a large number of factors, and multivariate analysis allows us to snapshot the obtained results from multiple directions, thereby enabling us to have more comprehensive view than does univariate analysis.

Additionally, we conducted a systematic review of studies from the last 20 years investigating metabolite differences between schizophrenia or psychotic disorder subjects and control subjects.

## 2 METHODS

### 2.1 Subjects

This study was approved by the Ethical Committee of Kobe University Graduate School of Medicine. All the participants were of Japanese descent and were recruited in the Hyogo prefecture, Japan. We obtained written informed consent after describing the study to the subjects. The psychiatric assessment of each participant was performed as previously described.^9^, ^10^

As recommended by the Diagnostic and Statistical Manual of Mental Disorders, 4th Edition (DSM‐IV) criteria, at least two psychiatrists diagnosed the patients with schizophrenia. The control group consisted of healthy volunteers. None of the control subjects had any present, past, or family (first‐degree relatives) histories of psychiatric disorders or substance abuse excluding nicotine dependence. We conducted interviews and screening for psychiatric disorders on all the control subjects.

Demographic and clinical characteristics are shown in Table 1. The subjects consisted of 20 unrelated patients with chronic schizophrenia (SCZc) and 20 unrelated healthy controls (CTL). Additionally, we took blood samples from 20 patients who were admitted due to acute psychosis, once at the time of admission (SCZa‐Acute) and once just before being discharged (SCZa‐Remission).

**TABLE 1:** Demographic and clinical characteristics

	CTL	Schizophrenia	On discharge (remission state)	Chronic schizophrenia	*P*‐value
		On admission (acute psychotic state)			
Number	20	20		20	
Sex (M/F)	10/10	11/9		10/10	0.935^a^
Age (years)	41.8 ± 12.2	42.5 ± 12.7		43.4 ± 8.8	0.911^b^
Onset (years)		26.9 ± 12.5		22.1 ± 5.3	0.123^b^
Duration (years)		15.6 ± 13.4		21.3 ± 10.8	0.147^b^
CP value		347.5 ± 447.9	875.6 ± 446.8	1140.3 ± 908.2	0.00103^b^
WBC		6045 ± 1705	5750 ± 1662	5855 ± 1320	0.835^b^
GAF		27.1 ± 10.3	59.4 ± 9.8	39.3 ± 8.1	<0.0001^b^
BPRS		61.7 ± 13.1	33.0 ± 12.8	46.2 ± 15.2	<0.0001^b^

Abbreviations: BPRS, Brief Psychiatric Rating Scale; GAF, global assessment of functioning.

^a^ *P*‐value was calculated using Chi‐squared test between the schizophrenia and control groups.

^b^ *P*‐value was calculated using Student's *t*‐test between the schizophrenia and control groups.

### 2.2 Measurement of metabolites

Metabolome measurements were carried out through a facility service at Human Metabolome Technologies Inc, Tsuruoka, Japan.

Serum samples were collected from patients with schizophrenia and control subjects, and stored at −80°C. For capillary electrophoresis time‐of‐flight mass spectrometry (CE‐TOFMS), 50 µL of serum was added to 450 µL of methanol containing internal standards (Solution ID: H3304‐1002, Human Metabolome Technologies) at 0°C in order to inactivate enzymes. The extract solution was thoroughly mixed with 500 µL of chloroform and 200 µL of Milli‐Q water and centrifuged at 2300 × g and 4°C for 5 min. The 350 µL of upper aqueous layer was centrifugally filtered through a Millipore 5‐kDa cutoff filter to remove proteins. The filtrate was centrifugally concentrated and re‐suspended in 50 µL of Milli‐Q water for CE‐MS analysis.

For liquid electrophoresis time‐of‐flight mass spectrometry (LC‐TOFMS), 500 µL of serum was added to 1500 µL of 1% formic acid/acetonitrile containing internal standard solution (Solution ID: H3304‐1002, Human Metabolome Technologies, Inc) at 0°C in order to inactivate enzymes. The solution was thoroughly mixed and centrifuged at 2300 × g and 4°C for 5 min. The supernatant was filtrated using Hybrid SPE phospholipid 55261‐U (Supelco) to remove phospholipids. The filtrate was desiccated and then dissolved with 100 µL of isopropanol/Milli‐Q for LC‐MS analysis.

### 2.3 Statistics

The collected data were imported to SIMCA software v.16.0.2 (Sartorius Stedim Data Analytics AB) for multivariate analysis. None of the samples were dropped from the analysis.

First, we carried out principal component analysis (PCA) to examine how all the metabolites differed among the four groups of interest (CTL, SCZa‐Acute, SCZa‐Remission, and SCZc). Score plots of PCA models consisted of two synthetic variables: principal component PC1 (the component explaining maximum variance in the data) and PC2 (the component explaining the second greatest variance in the data, orthogonal to PC1).

Next, we performed orthogonal partial least squares discriminant analysis (OPLS‐DA) to compare the two groups, CTL and SCZc. The R2 (cumulative) and Q2 (cumulative) statistics were calculated for this OPLS‐DA model. R2 represents the percentage of variation explained by the model (the goodness of fit), whereas Q2 indicates the predictive ability of the model.^7^, ^11^

S‐plots of this model were used to see which metabolites help in strongly differentiating these two groups. We checked the significance of the metabolites selected by OPLS‐DA using the non‐parametric Mann‐Whitney *U*‐test (two‐tailed) in R.

Finally, we used the OPLS regression model to compare the metabolites of the paired samples of SCZa‐Acute and SCZa‐Remission. This is called OPLS‐effect projection (OPLS‐EP) and was used to test which metabolites contribute most to differentiating the acute and remission states of the same patients. For metabolites selected by OPLS‐EP, we performed the paired non‐parametric Wilcoxon signed rank test (two‐tailed) in R to assess the changes between two states of the same patients.

Principal component analysis and OPLS‐DA were performed with pareto‐scaled datasets and OPLS‐EP was performed with unit variance (UV) scaled datasets. We performed this analysis similar to previous studies.^12^, ^13^, ^14^ We considered *P* < .05 as statistically significant for all statistical analyses. However, since we have 378 comparisons, we used Bonferroni correction to obtain the adjusted (α = 0.05/378 ≒ 0.000132). All the univariate analyses of our data were performed using R Version 4.0.2 (R Core Team, Vienna, Austria; https://www.R‐project.org).

### 2.4 Systematic review for possible biomarkers

We conducted a systematic search according to PRISMA guidelines.^15^

**FIGURE 1:**
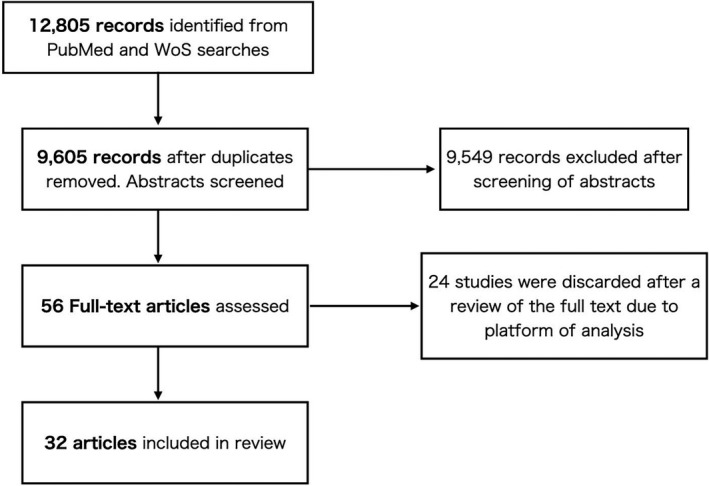
Flow chart of selection process. WoS, Web of Science

We used the following search terms by referring to a previous review^3^: “(schizophreni? OR psychosis OR “at risk mental state” OR “at‐risk mental state” OR ARMS OR “ultra‐high risk” OR UHR) AND (metabolom* OR metabolite? OR lipidom* OR lipid? OR biomarker? OR “biological marker?” OR “biological signature?”)” in the two databases, PubMed and Web of Science. The search was restricted to English language journal articles with human subjects, published between January 2000 and July 2020. There were 12 805 records identified through database searching, and 9653 remained after removing duplicates. Titles and abstracts of the 9653 articles were independently screened by at least two independent reviewers (S Kim and Y Shinko) for eligibility. After screening of abstracts, 56 records reached inclusion criteria while 24 studies were discarded after a review of the full text due to platform of analysis. As a result, 32 articles to be included in the review (Figure 1).

Abstracts were screened using the following criteria for inclusion/exclusion: only experimental published papers were included; reviews and meta‐analyses were excluded. Papers were included if they measured any biofluid metabolite levels in humans with schizophrenia spectrum disorders (eg, schizophrenia, schizoaffective disorders, and first‐episode psychosis) or those at risk of developing schizophrenia (eg, at‐risk mental state subjects and those with a family history of the disorder) and compared subjects to a healthy control group. To focus on studies with comprehensive analysis, we only included studies using proton NMR or MS and excluded studies using other techniques (general assay, enzyme‐linked immunosorbent assay, etc). Papers were excluded if: (a) objective disorders were other disorders, such as mood disorder, drug‐induced psychosis, or dementia, (b) compared subjects to the patients in a different state (ie, measured the relationship between metabolite levels and symptom severity or the effects of antipsychotic medications), or (c) focused on metabolites measured in postmortem brain tissue or magnetic resonance spectroscopy.

We summarized only the metabolites that showed significant changes and did not include those without significant differences.

## 3 RESULTS

### 3.1 Metabolomics data of schizophrenia patients and control subjects using CE‐TOFMS and LC‐ TOFMS

#### 3.1.1 Principal component analysis

**FIGURE 2:**
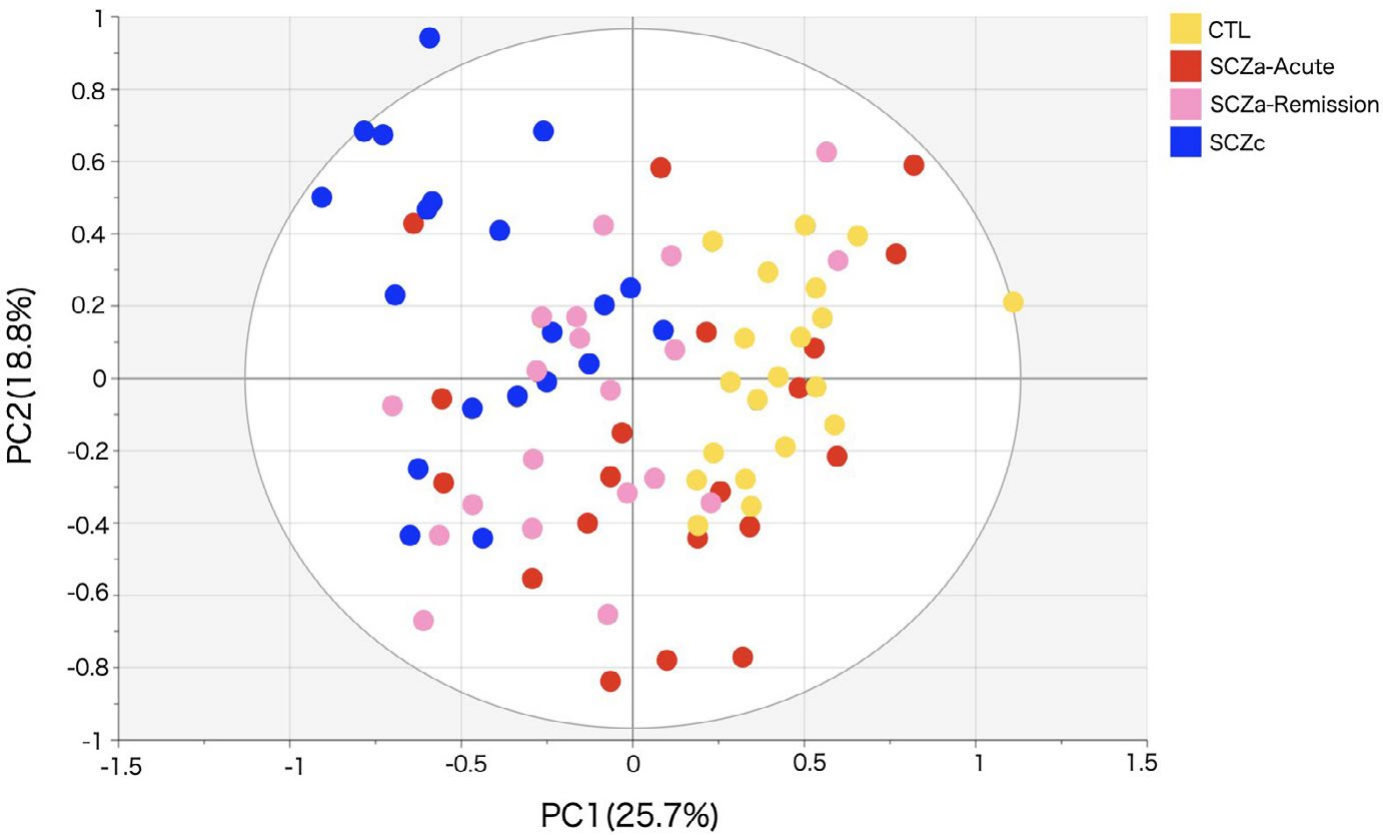
Principal component analysis scatter plot. The four groups are presented by different colors (yellow, CTL; red, SCZa‐Acute; pink, SCZa‐Remission; blue, SCZc)

A total of 378 metabolites were detected in the serum of the participants. The PCA score plot of PC1 and PC2 showed reasonably clear independent groupings (Figure 2). The scores of PC1, PC2, and the residuals were 25.7%, 18.8%, and 55.5%, respectively.

The CTL and SCZc groups were clearly separated from each other, while SCZa‐Acute and SCZa‐Remission groups were distributed between these two groups. The distribution of SCZa‐Remission was similar to, but narrower than, that of SCZa‐Acute.

#### 3.1.2 Comparison of control subjects and chronic schizophrenia patients

**FIGURE 3:**
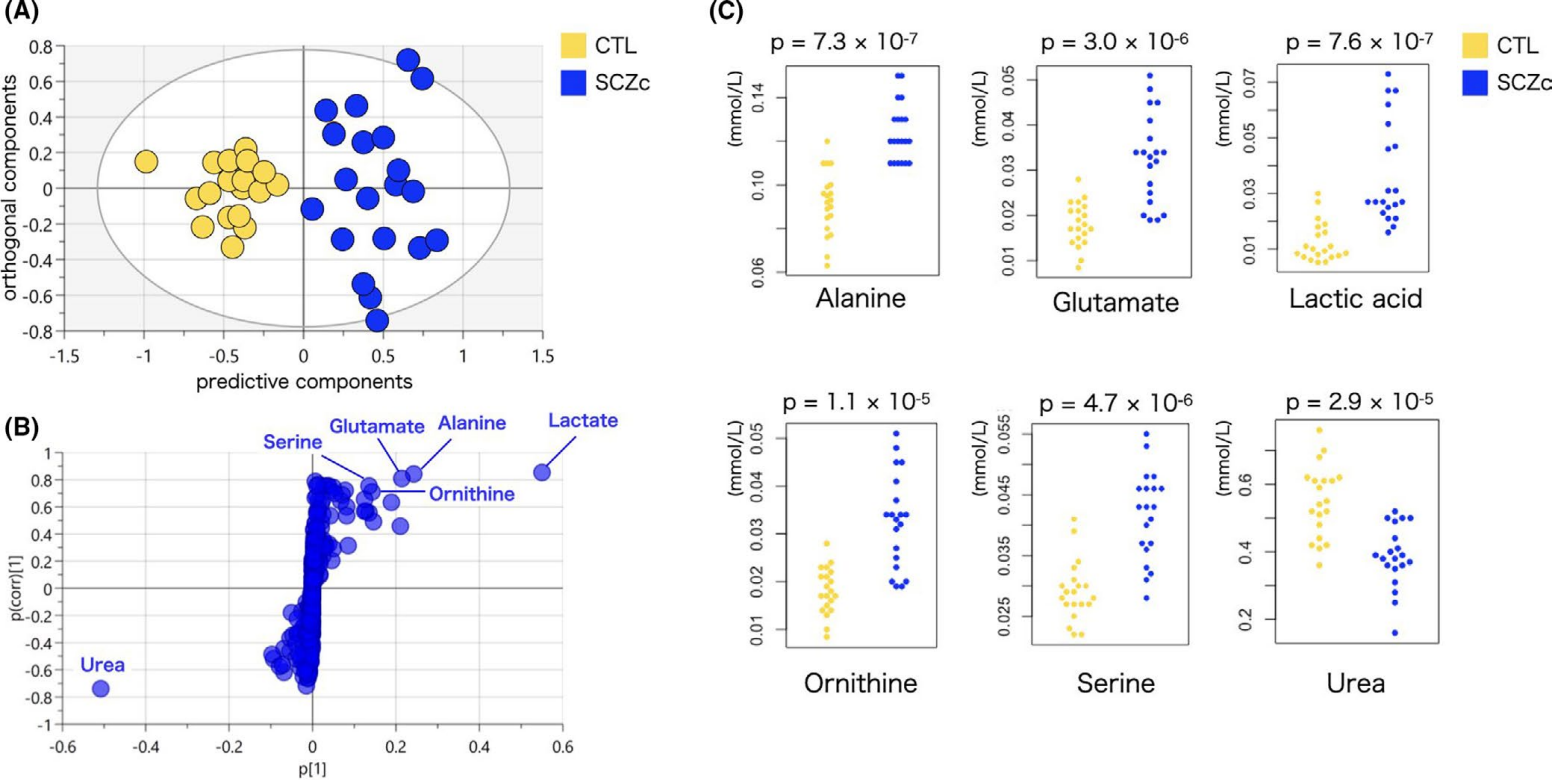
Overview of the comparison between patients with chronic schizophrenia (SCZc) and control group (CTL). A, OPLS‐DA score plot (R2(cum) = 0.79, Q2(cum) = 0.76, 2 components); OPLS‐DA score plots in the predictive (*x*‐axis) and orthogonal (*y*‐axis) components of metabolomic data. Separation of groups is maximized along the predictive component, while the orthogonal component accounts for intra‐group variability. B, S‐plot. p(corr)[1] and p[1] are the correlation coefficient and contribution coefficient vectors of the predictive component of the OPLS‐DA model. Left and right correspond to panel (A), ie, substances with positive p[1] indicate higher concentration in SCZc, C, The results of non‐parametric Mann‐Whitney U‐test (two‐tailed) between SCZc and CTL groups

In the score plot of OPLS‐DA with two components, both R2 and Q2 were statistically significant (R2 = 0.79, Q2 = 0.76) and the two groups SCZc and CTL were clearly separated (Figure 3A).

The resulting S‐plot is shown in Figure 3B. The horizontal axis (ie, the loading) describes the magnitude of each variable, and the vertical axis represents the reliability of each variable in the OPLS‐DA data. Metabolites that exceeded the threshold of 0.70 for p(corr)[1] values and 0.1 for p[1] values are labeled in Figure 3B, and considered as significantly different between the two classes. Full data sets are shown in Table S1.

In total, six unique metabolites (alanine, glutamate, lactic acid, ornithine, serine, and urea) were identified to be responsible for class separation. We performed the non‐parametric Mann‐Whitney *U*‐test (two‐tailed) on these six selected metabolites and observed significant differences in all these metabolites (*P* < .000132) (Figure 3C).

**FIGURE 4:**
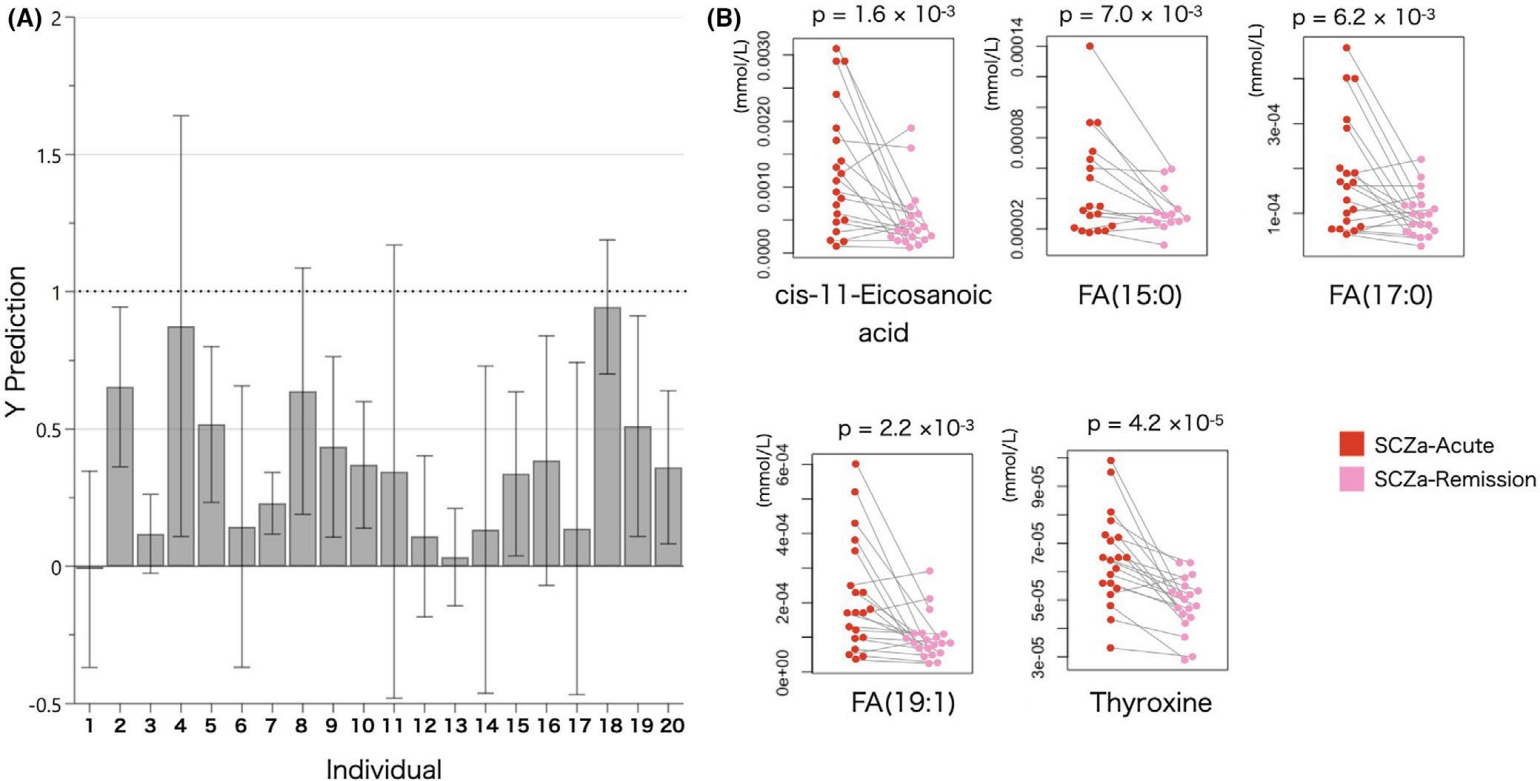
Overview of the comparison between schizophrenia patients with acute psychosis (SCZa‐Acute) and in remission state (SCZa‐Remission). A, OPLS‐EP loading plot of metabolites profile changes after remission compared with the acute phase; the dotted line (Y prediction = 1) indicates the target value for the OPLS‐EP model. The magnitude of the projected effect is given by the height of the corresponding grey bar. Deviations from the value 1 for a specific patient indicate a larger (>1) or smaller (<1) metabolic effect of SCZa‐Remission compared with SCZa‐Acute in the model direction (metabolic profile). B, The results of a paired non‐parametric Wilcoxon signed rank test (two‐tailed) between SCZa‐Acute and SCZa‐Remission

We also performed a multivariate logistic regression analysis to determine whether these six metabolites, sex, and age are biomarkers for schizophrenia. We first tested the six metabolites individually and found that the AUCs ranged from 0.807 to 1.0 (Figure S1A–F). In addition, the ROC analysis including sex and age as fixed factors without the metabolites showed low accuracy (AUC = 0.569 [95% CI = 0.383–0.755]) (Figure 4G). This indicates that these metabolites improve the potential to differentiate the chronic schizophrenia and control groups.

In addition, Spearman's correlation analysis was performed to determine the effect of antipsychotic drugs on the differences in the values of these metabolites. After applying Bonferroni corrections (α = 0.05/6 ≒ 0.0083), we observed no significant correlation between the difference in each metabolite and the chlorpromazine equivalents (CP) (Figure S2A‐F).

We also performed a regression analysis using a generalized linear model with gamma distribution and log link, in which serum metabolite level was the response variable, and age, sex, and antipsychotic dose were the explanatory variables (Table S2). After applying Bonferroni corrections (α = 0.05/6 ≒ 0.0083), we observed no significant correlation.

#### 3.1.3 Comparison of acute phase and remission phase

An OPLS model with two components yielded R2 = 0.66 and Q2 = 0.40, and moderately separated SCZa‐Acute and SCZa‐Remission. In the effect projection plot, most patients in remission state showed smaller (<1) metabolic effect compared with their acute state in the model direction of metabolic profile (Figure 4A). We divided the absolute value of the difference in the loading of each metabolite in each individual by the absolute value of the confidence interval, and considered the effect projection to be significant in order of its divided value. As a result, we selected the top five substances as unique metabolites, three odd chain fatty acids, fatty acid (15:0), (17:0), and (19:1), one mono‐unsaturated fatty acid (cis‐11‐eicosenoic acid), and thyroxine as being most responsible for class separation. Full data sets are shown in Table S3.

Levels of these five metabolites were significantly higher in SCZa‐Acute than SCZa‐Remission according to Wilcoxon signed rank test (*P* < .05) (Figure 4B). In addition, Spearman's correlation analysis was performed to determine the effect of antipsychotic drugs on the differences in the values of these metabolites. To estimate the average daily dosage the patients took between acute psychosis and remission state, and to see if there is the correlation between the change of antipsychotic dosage and metabolites, we tested for both the average and the difference of the chlorpromazine equivalents (CP) of SCZa‐Acute and SCZa‐Remission. After applying Bonferroni corrections (α = 0.05/5 = 0.01), we observed no significant correlation between the difference in each metabolite and the average of CP (Figure S3A‐E), or the difference of CP (Figure S4A‐E).

### 3.2 Systematic review on metabolite biomarkers

**FIGURE 5:**
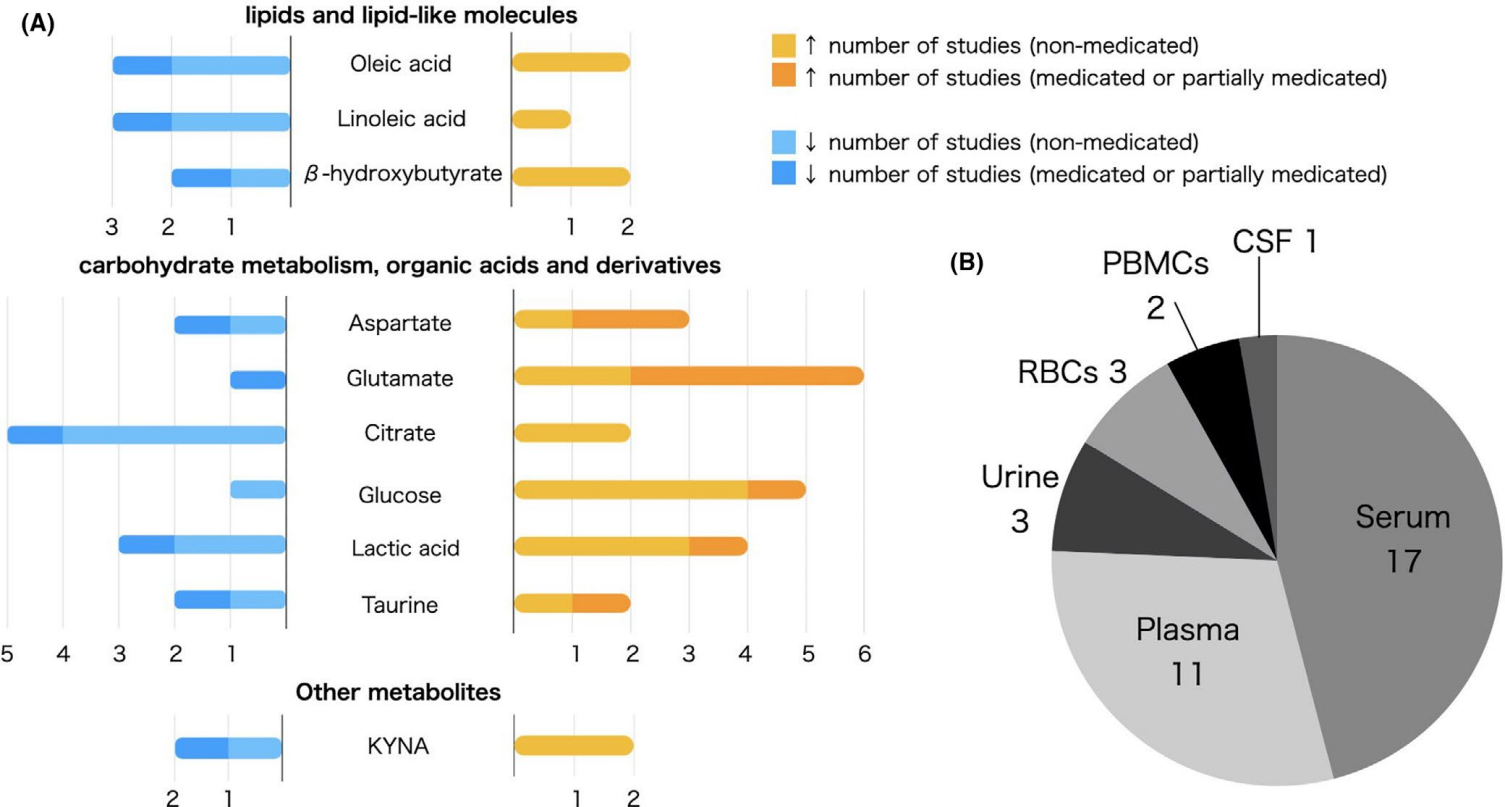
Summary of the systematic review of alterations in metabolites in schizophrenia patients. A, The metabolites reported in more than four reports. Bars represent the number of studies with reported differences. B, Biofluids analyzed in reviewed studies (Arc lengths of slices of the pie chart correspond to the number of studies

We reviewed and summarized 33 reports including our data (Tables S4‐S6). The metabolites reported to be significant in four or more reports were summarized (Figure 5). We divided results into sections based on metabolite classes: (1) lipids and lipid‐like molecules (including fatty acids, steroids, and other lipid‐like molecules), (2) carbohydrate metabolites, organic acids, and derivatives, and (3) other metabolites.

#### 3.2.1 Lipids and lipid‐like molecules

Significant changes in fatty acids, such as oleic acid,^16^, ^17^, ^18^, ^19^, ^20^ linoleum acid,^17^, ^18^, ^19^, ^21^ and ketone‐body β‐hydroxybutyrate,^19^, ^21^, ^22^ were reported in four or more studies. However, the results of each metabolite were inconsistent across these studies.

#### 3.2.2 Carbohydrate metabolites, organic acids, and derivatives

Glucose,^18^, ^19^, ^22^, ^23^ lactic acid,^18^, ^19^, ^22^, ^23^ citrate,^18^, ^19^, ^22^, ^23^ and the amino acids aspartate,^18^, ^19^, ^24^, ^25^, ^26^ glutamate,^19^, ^21^, ^25^, ^26^, ^27^, and taurine^22^, ^25^, ^28^, ^29^ were reported to be significantly different in schizophrenia patients in four or more reports. Increase in glucose was reported in several papers and was not necessarily accompanied with antipsychotic use as four of these studies were conducted on drug‐free patients.^18^, ^19^, ^22^, ^23^

We also observed a trend that citrate decreased^18^, ^19^, ^22^, ^24^ and glutamate increased^19^, ^21^, ^26^, ^27^ in psychotic patients. However, the differences in aspartate, taurine, and lactic acid were inconsistent across studies.

#### 3.2.3 Other metabolites

Changes in kynurenic acid were reported in four reports.^30^, ^31^, ^32^, ^33^ The differences were inconsistent across studies.

## 4 DISCUSSION

We observed significant differences in the levels of several amino acids, lactic acid, and urea between CTL and SCZc groups in our case‐controlled experiments. Moreover, we found significant differences in several fatty acids and thyroxine between the same individuals during the acute and remission phases (SCZa‐Acute and SCZa‐Remission groups). As these substances were not correlated with the amount of antipsychotic medications, we concluded that the metabolites identified in the first set may be biomarkers of schizophrenia while the metabolites in the second set may be state markers of acute psychosis.

Glutamate is an amino acid known to be a major excitatory neurotransmitter in the brain.^27^ Several studies report dysregulation of the glutamatergic system in schizophrenia.^34^, ^35^ As summarized in Section 3.2.2, recent studies showed trends toward increased plasma glutamate levels in patients with psychotic disorders consistent with our data. However, glutamate levels could also increase due to improvement of symptoms or use of antipsychotic medicines^34^, ^36^, ^37^ and benzodiazepines.^35^ Hence, more studies are needed to fully reveal the relationship between medication and peripheral glutamate concentration.

Serine and alanine are categorized as non‐essential amino acids that can be synthesized in the body. D‐serine is an intrinsic D‐amino acid synthesized in the central nervous system from L‐serine by serine racemase, whereas D‐alanine is an extrinsic D‐amino acid found in the gut microbiome.^38^ Both of these D‐amino acids are known to be co‐agonists of NMDA receptors and promote glutamatergic activation of this receptor. Some randomized controlled trials showed that D‐serine^39^ and D‐alanine^40^ improved the symptoms of patients with schizophrenia. On the other hand, recent studies reported increased levels of peripheral serine^19^ and alanine^22^ in schizophrenia patients, consistent with our results. Hashimoto et al^41^ suggested abnormalities in the syntheses or metabolism of L‐serine in schizophrenia patients,however, there is no consensus on this mechanism yet.

Ornithine is an amino acid component within the urea cycle, which plays a significant role in the human body. Recent studies reported higher blood concentration of ornithine in schizophrenia patients, which is consistent with our observations^25^, ^42^. He et al^42^ suggested that dysfunction of ODC1 could increase ornithine levels, though this mechanism is yet to be fully understood.

On the other hand, urea, which is also a metabolite of the urea cycle, was significantly lower in patients with schizophrenia. No previous studies have reported a relationship between schizophrenia and blood urea concentration. Zhai et al^43^ reported that antipsychotic use significantly decreased blood urea level suggesting that this may be an effect of antipsychotic medication. More studies are needed to understand this mechanism.

Lactic acid is an organic acid used as a marker for mitochondrial diseases. In such disorders, metabolism relies on extra‐mitochondrial glycolysis and lactic acids accumulate since they are not metabolized.^44^ Mitochondrial dysfunction in schizophrenia has been previously reported^45^ and could be related to lactic acid accumulation. Both increased and decreased lactic acid levels were reported in studies of patients with schizophrenia^18^, ^19^, ^22^, ^23^ and our data were consistent with some reports.^18^, ^19^, ^22^ However, the potential antipsychotic effect of mitochondrial function should not be ignored.^46^

Various types of odd‐chain fatty acids are seen in bacteria, fungi, plants, and animals. Bacteria generally contain odd‐numbered fatty acids, such as C15, C17, or C19.^47^ While these fatty acids have only recently received attention, Yang et al^20^ reported significant changes in odd‐chain fatty acids in schizophrenia patients, partly consistent with our data. These abnormalities may imply the disturbance of gut microbiota in patients with psychosis as many recent reports have suggested.^48^

Cis‐11‐Eicosenoic acid (gondoic acid) is a monounsaturated omega‐9 fatty acid found in a variety of plant oils and nuts. While not many studies have examined this fatty acid, our data were consistent with Yang et al.^19^ Our findings could suggest systematic alternations in glycogenolysis and lipid metabolism in patients with acute psychosis, similar to suggestions by previous studies.

Thyroxine, the hormone produced and released by the thyroid gland, has also recently attracted attention in relation to schizophrenia. Although we did not find any consistent reports of thyroxine in our systematic review, our metabolomics study indicates dysregulation of the pituitary‐thyroid axis, which could be related to major neurosignaling systems in psychosis as suggested by Santos et al.^49^

Following a large‐scale systematic review in 2018,^3^ several studies have been conducted in this field. Some reported differences in metabolites, such as glutamate and lactic acid, were consistent with our findings, while we did not observe significant differences in other previously reported metabolites, such as some fatty acids, citric acid, and kynurenine. Some studies reported contradictory findings between different samples from the same patients.^19^, ^22^ Yang et al^19^ suggested that this could be due to different metabolic/excretion rates in different biological compartments. Hence, more comparative studies, such as the review on cerebrospinal fluid and blood by Quinones and Kaddurah‐Daouk,^50^ are needed to map central and peripheral changes in psychotic disorders.

Nuclear magnetic resonance and MS are the most common analytical techniques used for biofluid metabolite analysis. While NMR is quantitative and does not require extra steps for complicated sample preparation, MS provides better sensitivity and selectivity platforms.^51^ MS is usually used with other separation techniques, such as CE, gas chromatography, and LC to achieve better detection. Since each techniques has its own limits, some studies combine two analytical methods.^19^, ^25^ Similarly, we combined CE‐TOFMS and LC‐TOFMS in our study. With further improvements in such technique which would allow us to analyze a wider range of compounds with higher qualitative reliability, we would be able to collect more reliable data to identify possible biofluid markers in schizophrenia.

One of the biggest challenges in this field is the difficulty in excluding biases due to effects of antipsychotic medication, diet, activities, and hospitalization. Although some studies were conducted for drug‐naive or drug‐free patients with psychosis, those studies focus on patients with comparatively mild symptoms. It is ethically challenging to obtain the data of patients with severe symptoms without antipsychotic medications as they need prompt and proper treatment the most. Data on the effects of medications and environmental factors on peripheral metabolites, such as those reported by Suvitaival et al,^52^ would be important to exclude environmental biases and accurately identify biomarkers based on pathology.

The other big challenge we face is that schizophrenia may not be a single disease, but rather a syndrome, which is affected by a wide variety of genetical and biological variables.^53^ Inconsistent results of metabolites in schizophrenia could be due to these diverge pathologies. Furthermore, if we could identify metabolomic effects of psychotic disorders, medication, and acute or remission state of psychosis more correctly, they might also reveal pathologies of schizophrenia, which is still far from being fully understood.

As this research field is rapidly expanding, more studies can shed further light on these questions.

One of the major limitations of our study was that the sample size was not large with 20 participants for each group. Moreover, the participants in our study were all Japanese, which limits the extrapolation of our findings to a more general population. Additionally, the scores of PC1 and PC2 were 25.7 and 18.8, which may not be considered robustly significant. We suggest that it is difficult to degrade all the variables into two components since there were too many variables and various directions.

Furthermore, there could be biases due to hospitalization between each group. We could not exclude these biases because all the patients included in the study were severely ill and needed to be treated in a hospitalized setting.

Nevertheless, we believe our study is an important primer for further studies that could substantiate and expand our findings.

## CONFLICT OF INTEREST

The authors declare that they have no conflict of interest.

## AUTHOR CONTRIBUTION

Saehyeon Kim: Conceptualization, Methodology, Investigation, Formal analysis, Writing – original draft. Satoshi Okazaki: Methodology, Investigation, Formal analysis, Writing – original draft. Ikuo Otsuka: Formal analysis, Data curation, Writing – review & editing. Yutaka Shinko: Formal analysis, Data curation. Tadasu Horai, Naofumi Shimmyo, Takashi Hirata, Naruhisa Yamaki, Takaki Tanifuji: Data curation. Shuken Boku: Supervision, Writing – review & editing. Ichiro Sora: Supervision. Akitoyo Hishimoto: Conceptualization, Methodology, Investigation, Formal analysis, Writing – original draft, and Writing – review & editing.

## APPROVAL OF THE RESEARCH PROTOCOL BY AN INSTITUTIONAL REVIEWER BOARD

The protocol for this research project has been approved by a suitably constituted Ethics Committee of the institution and it conforms to the provisions of the Declaration of Helsinki.

## INFORMED CONSENT

All informed consent was obtained from the subjects or guardians.

## REGISTRY AND THE REGISTRATION NO. OF THE STUDY

n/a

## ANIMAL STUDIES

n/a

## Supporting information

Figure S1

Figure S2

Figure S3

Figure S4

Table S1

Table S2

Table S3

Table S4–S6

## Data Availability

The data that supports the findings of this study are available in the [Link], [Link], [Link], [Link], [Link], [Link], [Link], [Link] of this article.
