# Supplementary figures and images for: Searching for biomarkers in schizophrenia and psychosis: Case‐control study using capillary electrophoresis and liquid chromatography time‐of‐flight mass spectrometry and systematic review for biofluid metabolites
Source: Neuropsychopharmacol Rep. 2021 Dec 8;42(1):42–51. doi: 10.1002/npr2.12223 (PMC8919119; doi:10.1002/npr2.12223)

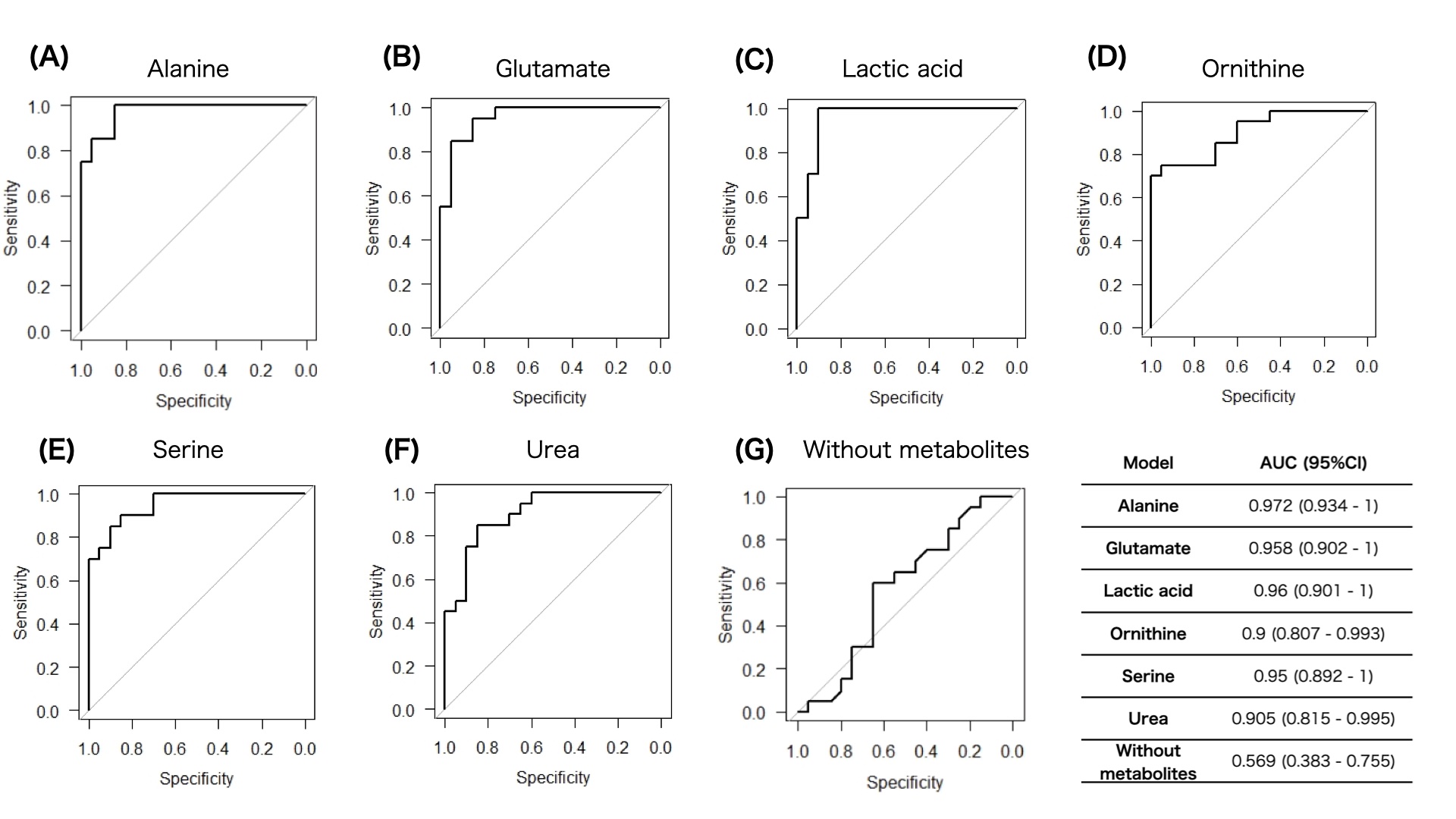

Supplement: Supplementary file 1 — Figure S1 [file NPR2-42-42-s001.jpg]

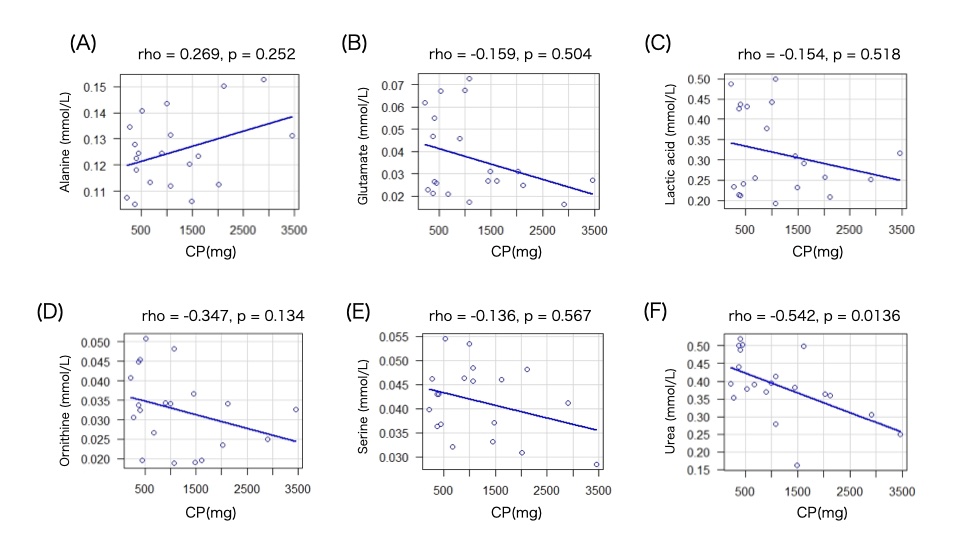

Supplement: Supplementary file 2 — Figure S2 [file NPR2-42-42-s006.jpg]

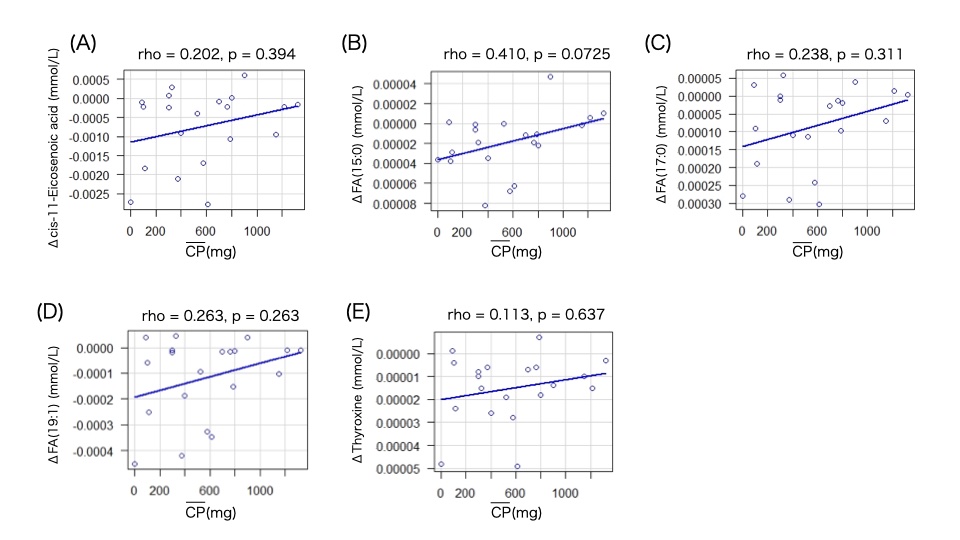

Supplement: Supplementary file 3 — Figure S3 [file NPR2-42-42-s007.jpg]

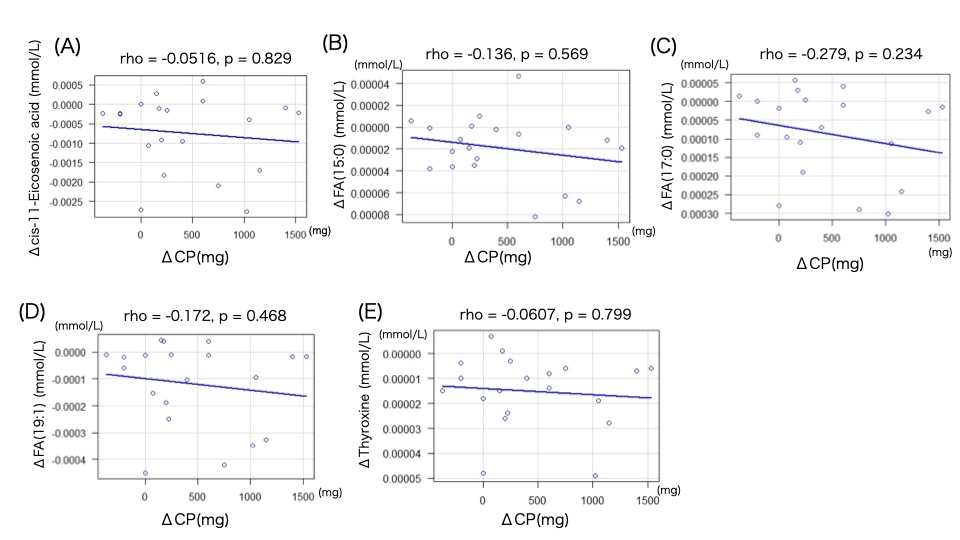

Supplement: Supplementary file 4 — Figure S4 [file NPR2-42-42-s005.jpg]
