# Supplementary material for: Searching for biomarkers in schizophrenia and psychosis: Case‐control study using capillary electrophoresis and liquid chromatography time‐of‐flight mass spectrometry and systematic review for biofluid metabolites
Source: Neuropsychopharmacol Rep. 2021 Dec 8;42(1):42–51. doi: 10.1002/npr2.12223 (PMC8919119; doi:10.1002/npr2.12223)
Supplement: Supplementary file 6 — Table S2 [file NPR2-42-42-s004.docx]

| Variable | Estimate | SE | *t value* | *p*-value |
| --- | --- | --- | --- | --- |
| Alanine |  |  |  |  |
| CP | 4.65×10^-5^ | 3.32×10^-5^ | 1.405 | 0.179 |
| Sex | 0.0173 | 0.0561 | 0.308 | 0.762 |
| Age | -7×10^-4^ | 0.003 | -0.228 | 0.822 |
| Glutamate |  |  |  |  |
| CP | -2.24×10^-4^ | -1.26×10^-4^ | -1.769 | 0.0959 |
| Sex | -0.322 | 0.214 | -1.502 | 0.153 |
| Age | 0.0182 | 0.0117 | 1.55 | 0.14 |
| Lactic acid |  |  |  |  |
| CP | -1×10^-4^ | 8.4×10^-5^ | -1.202 | 0.247 |
| Sex | -0.257 | 0.142 | -1.811 | 0.0889 |
| Age | 0.0138 | 0.00777 | 1.775 | 0.095 |
| Ornithine |  |  |  |  |
| CP | -1.06×10^-4^ | 8.86×10^-5^ | -1.19 | 0.249 |
| Sex | -0.0949 | 0.150 | -0.633 | 0.536 |
| Age | 0.00680 | 0.00820 | 0.829 | 0.42 |
| Serine |  |  |  |  |
| CP | -6.6×10^-5^ | 5.03×10^-5^ | -1.31 | 0.209 |
| Sex | 0.0864 | 0.0853 | 1.013 | 0.326 |
| Age | -0.0052 | 0.0047 | -1.11 | 0.282 |
| Urea |  |  |  |  |
| CP | -1.32×10^-4^ | 6.17×10^-5^ | -2.14 | 0.0481 |
| Sex | 0.113 | 0.104 | 1.082 | 0.295 |
| Age | -0.001 | 0.00571 | -0.189 | 0.853 |

**Supplementary Table s2.** Regression analyses using a generalized linear model of the serum metabolite levels in chronic schizophrenia group.

Regression analyses were performed using a generalized linear model with gamma distribution and log link, in which serum metabolite level was the response variable, and each age, sex, and antipsychotic dose were the explanatory variables in schizophrenia groups. SE, standard error. CP, chlorpromazine equivalents. Dummy variables were used as follows: sex, male = 0 and female = 1.
