# Supplementary material for: Searching for biomarkers in schizophrenia and psychosis: Case‐control study using capillary electrophoresis and liquid chromatography time‐of‐flight mass spectrometry and systematic review for biofluid metabolites
Source: Neuropsychopharmacol Rep. 2021 Dec 8;42(1):42–51. doi: 10.1002/npr2.12223 (PMC8919119; doi:10.1002/npr2.12223)
Supplement: Supplementary file 8 — Table S4–S6 [file NPR2-42-42-s008.docx]

**Table s4: Lipids and lipid-like molecules found at abnormal levels in subjects with schizophrenia**

| **Metabolite** | **Reference** | **Platform** | **Biofluid** | **Difference (SCZ v control)** | **Subjects** | **Medication** | **P-value** |
| --- | --- | --- | --- | --- | --- | --- | --- |
| **Acylcarnitine & Fatty acids** | | | | | | | |
| **Acylcarnitine** | | | | | | | |
| AC (C3) | (Kriisa et al., 2017) | LC-MS | serum | ↓ | FEP before treatment (N=38); HC (N=37) | - | p < 0.05 |
| ACs (C14:1, C16, C18.1) | (Kriisa et al., 2017) | LC-MS | serum | ↑ | FEP before treatment (N=38); HC (N=37) | - | p < 0.05 |
| AC (C18:2) | (Yan et al., 2018) | LC-MS | plasma | ↓ | SCZ (N=20, M=11); HC (N=29,M-18) | - | p= 2.49 × 10^-5^ |
| ACs (C4-OH, C16:1) | (Cao et al., 2019) | LC-MS | plasma | ↑ | DF SCZ (N=225; 90M; DF ≥ 4 weeks (40 FE); HC (N=175) | - | q < 0.001 |
| ACs (C3, C8, C10, C10:1, C10:2, C12, C14:1-OH, C14:2, C14:2-OH) | (Cao et al., 2019) | LC-MS | plasma | ↓ | DF SCZ (N=225; 90M; DF ≥ 4 weeks (40 FE); HC (N=175) | - | q < 0.001 |
| **Fatty Acids** | | | | | | | |
| **Saturated Fatty Acids (SFAs)** | | | | | | | |
| Octanoic acid  C8:0 | (Liu et al., 2014) | GC-MS | PBMCs | ↑ | SCZ (N=45; 18M; 19 FE DN, 26 med; age = 33.2 ± 12.9) v HC (N=50; 22M; age = 37.3 ± 8.7) | + | p =1.39 × 10^−3^ |
| Nonanoic acid  C9:0 | (Koike et al., 2014) | CE-TOFMS | plasma | ↓ | 1^st^ set: FE SCZ (N= 18; 13M; most med, 2 DN; 4 disorganised, 7 paranoid, 3 SFD, 2 delusional disorder, 2 PD NOS; age = 23.2 ± 5.4) v HC (N=14; 11M; age = 25.7 ± 6.1)  2^nd^ set: FE SCZ (N=12; 4 disorganised, 3 paranoid, 3 SFD, 2 PD NOS; 2 DN; age = 24.6 ± 7.1) v HC (N=24 HC; 10M; age = 26.1 ± 2.6) | - | p = 0.025 (1st set)  p < 3 x 10^-4^ (2nd set) |
| Tetradecanoic acid  C14:0 | (Yang et al., 2013) | GC-TOFMS, ^1^H NMR | serum | ↑ | SCZ (N=62; 25M; age = 36.9 ± 11.9; DF ≥ 2 weeks, some FE) v HC (N=62; 25M; age = 36.9 ± 9.3) | - | p <10^-5^, q <0.01 |
| Hexadecanoic (palmitic) acid  C16:0 | (Xuan et al., 2011) | GC-MS | serum | ↓ | DF SCZ (N=18 ; 10M; age = 38 ± 15); HC (N=18 ; 10M; age = 41 ± 19) | - | p =0.0037 |
|  | (Yang et al., 2013) | GC-TOFMS, ^1^H NMR | serum | ↑ | SCZ (N=62; 25M; age = 36.9 ± 11.9; DF ≥ 2 weeks, some FE) v HC (N=62; 25M; age = 36.9 ± 9.3) | - | p <0.001, q <0.02 |
|  | (Yang et al., 2017) | LC-MS | serum | ↑ | SCZ (N=60; 24M; age = 37.2±12.0, DF) v HC (N=61; 25M; age = 36.9 ± 9.7) | - | p = 0.008 |
| 2-hydroxyethyl palmitate | (Liu et al., 2014) | GC-MS | PBMCs | ↑ | SCZ (N=45; 18M; 19 FE DN, 26 med; age = 33.2 ± 12.9) v HC (N=50; 22M; age = 37.3 ± 8.7) | + | p <0.05 |
| Heptadecanoic acid  C17:0 | (Al Awam et al., 2015) | FTIR, GC-MS | serum | ↓ | med SCZ (N=26; 20M; age = 37.3 ± 12.4) v HC (N=26; 20M; age = 37.0 ± 10.7) | + | p <0.01 |
| Octadecanoic (stearic) acid  C18:0 | (Schwarz et al., 2008) | LC-MS | RBCs | ↓ | FE DN SCZ (N = 7) v HC (N = 20) | - | p <0.005, q ≤0.002 |
|  | (Xuan et al., 2011) | GC-MS | serum | ↓ | DF SCZ (N=18 ; 10M; age = 38 ± 15); HC (N=18 ; 10M; age = 41 ± 19) | - | p <0.02 |
|  | (Yang et al., 2013) | GC-TOFMS, ^1^H NMR | serum | ↑ | SCZ (N=62; 25M; age = 36.9 ± 11.9; DF ≥ 2 weeks, some FE) v HC (N=62; 25M; age = 36.9 ± 9.3) | - | p <0.04, q <0.04 |
| Eicosanoic acid  C20:0 | (Yang et al., 2013) | GC-TOFMS, ^1^H NMR | serum | ↑ | SCZ (N=62; 25M; age = 36.9 ± 11.9; DF ≥ 2 weeks, some FE) v HC (N=62; 25M; age = 36.9 ± 9.3) | - | p <10^-5^, q <0.01 |
|  | (Al Awam et al., 2015) | FTIR, GC-MS | serum | ↓ | med SCZ (N=26; 20M; age = 37.3 ± 12.4) v HC (N=26; 20M; age = 37.0 ± 10.7) | + | p <0.001 |
| Lignoceric acid  C24:0 | (Yang et al., 2017) | LC-MS | serum | ↓ | SCZ (N=60; 24M; age = 37.2±12.0, DF) v HC (N=61; 25M; age = 36.9 ± 9.7) | - | p = 0.018 |
| **Monosaturated Fatty Acids (MUFAs)** | | | | | | | |
| Myristoleic acid  C 14:1 *cis*-9 | (Yang et al., 2017) | LC-MS | serum | ↑ | SCZ (N=60; 24M; age = 37.2±12.0, DF) v HC (N=61; 25M; age = 36.9 ± 9.7) | - | p = 0.003 |
| Palmitoleic acid  C 16:1 *cis*-9 | (Yang et al., 2017) | LC-MS | serum | ↑ | SCZ (N=60; 24M; age = 37.2±12.0, DF) v HC (N=61; 25M; age = 36.9 ± 9.7) | - | p = 9.23 × 10^-6^ |
| Oleic acid  C 18:1 *cis*-9 | (Schwarz et al., 2008) | LC-MS | RBCs | ↓ | FE DN SCZ (N=7) v HC (N=20) | - | p <0.005 |
|  | (Xuan et al., 2011) | GC-MS | serum | ↓ | DF SCZ (N=18 ; 10M; age = 38 ± 15); HC (N=18 ; 10M; age = 41 ± 19) | - | p <0.02 |
|  | (Yang et al., 2013) | GC-TOFMS, ^1^H NMR | serum | ↑ | SCZ (N=62; 25M; age = 36.9 ± 11.9; DF ≥ 2 weeks, some FE) v HC (N=62; 25M; age = 36.9 ± 9.3) | - | p <0.001, q <0.02 |
|  | (Al Awam et al., 2015) | FTIR, GC-MS | serum | ↓ | med SCZ (N=26; 20M; age = 37.3 ± 12.4) v HC (N=26; 20M; age = 37.0 ± 10.7) | + | p <0.05 |
|  | (Yang et al., 2017) | LC-MS | serum | ↑ | SCZ (N=60; 24M; age = 37.2±12.0, DF) v HC (N=61; 25M; age = 36.9 ± 9.7) | - | p = 7.9 × 10^-8^ |
| Cis-11-Eicosenoic acid  C 20:1 *cis*-11 | (Yang et al., 2017) | LC-MS | serum | ↑ | SCZ (N=60; 24M; age = 37.2±12.0, DF) v HC (N=61; 25M; age = 36.9 ± 9.7) | - | p = 7.9 × 10^-8^ |
| Erucic acid  C 22:1 *cis*-13 | (Yang et al., 2017) | LC-MS | serum | ↑ | SCZ (N=60; 24M; age = 37.2±12.0, DF) v HC (N=61; 25M; age = 36.9 ± 9.7) | - | p = 7.9 × 10^-8^ |
| Nervonic acid  C 24:1 *cis*-15 | (Yang et al., 2017) | LC-MS | serum | ↑ | SCZ (N=60; 24M; age = 37.2±12.0, DF) v HC (N=61; 25M; age = 36.9 ± 9.7) | - | p = 2.05 × 10^-8^ |
| **n-6 PUFAs** | | | | | | | |
| Linoleic acid  C18:2 *cis*-9,12 | (Schwarz et al., 2008) | LC-MS | RBCs | ↓ | FE DN SCZ (N=7) v HC (N=20) | - | p <0.005, q ≤0.002 |
|  | (Xuan et al., 2011) | GC-MS | serum | ↓ | DF SCZ (N=18 ; 10M; age = 38 ± 15); HC (N=18 ; 10M; age = 41 ± 19) | - | p <0.02 |
|  | (Yang et al., 2013) | GC-TOFMS, ^1^H NMR | serum | ↑ | SCZ (N=62; 25M; age = 36.9 ± 11.9; DF ≥ 2 weeks, some FE) v HC (N=62; 25M; age = 36.9 ± 9.3) | - | p <0.04, q <0.04 |
|  | (Fukushima et al., 2014) | LC-MS | serum | ↓ | med SCZ (N=25; 11M; age = 28.2 ± 4.4) v HC (N=27; 12M; age = 26.5 ± 5.6) | + | p =1.61 x 10^-5^ (survived Bonferroni) |
| Pentadecanoic acid  C15:0 | (Al Awam et al., 2015) | FTIR, GC-MS | serum | ↓ | med SCZ (N=26; 20M; age = 37.3 ± 12.4) v HC (N=26; 20M; age = 37.0 ± 10.7) | + | p <0.01 |
| Eicosadienoic acid  C20:2 *cis*-11,14 | (Yang et al., 2017) | LC-MS | serum | ↑ | SCZ (N=60; 24M; age = 37.2±12.0, DF) v HC (N=61; 25M; age = 36.9 ± 9.7) | - | p = 0.007 |
| Dihomo-γ-linolenic acid  ﻿C20:3 cis-8,11,14 | (Yang et al., 2017) | LC-MS | serum | ↑ | SCZ (N=60; 24M; age = 37.2±12.0, DF) v HC (N=61; 25M; age = 36.9 ± 9.7) | - | p = 0.013 |
| Cis-5.8.11.14-eicosapentaenoic acid  ﻿C20:4 cis-5,8,11,14 | (Yang et al., 2017) | LC-MS | serum | ↑ | SCZ (N=60; 24M; age = 37.2±12.0, DF) v HC (N=61; 25M; age = 36.9 ± 9.7) | - | p = 0.0038 |
| Arachidonic acid  C 20:4 | (Schwarz et al., 2008) | LC-MS | RBCs | ↓ | FE DN SCZ (N=7) v HC (N=20) | - | p <0.005 |
|  | (Fukushima et al., 2014) | LC-MS | serum | ↓ | med SCZ (N=25; 11M; age = 28.2 ± 4.4) v HC (N=27; 12M; age = 26.5 ± 5.6) | + | p <0.005 |
|  | (Wang et al., 2018) | LC-MS | serum | ↓ | SCZ (N=115; 51M; age = 29.0 (IQR 25.0-33.2); 88 DF ≥ 1 months, 27 FE) v HC (N=108; 37M; age = 30.0 (IQR 26.0-33.0)) | - | q = 4.77 × 10^-6^ |
| 20-carboxy-arachidonic acid | (Wang et al., 2018) | LC-MS | serum | ↑ | SCZ (N=115; 51M; age = 29.0 (IQR 25.0-33.2); 88 DF ≥ 1 months, 27 FE) v HC (N=108; 37M; age = 30.0 (IQR 26.0-33.0)) | - | q = 0.0045 |
| ﻿Cis-13,16-Docosahexaenoic acid  C22:2 *cis*-13,16 | (Yang et al., 2017) | LC-MS | serum | ↑ | SCZ (N=60; 24M; age = 37.2±12.0, DF) v HC (N=61; 25M; age = 36.9 ± 9.7) | - | p = 0.0046 |
| Docosatetraenoic acid  ﻿C22:4 *cis*-7,10,13,16 | (Yang et al., 2017) | LC-MS | serum | ↑ | SCZ (N=60; 24M; age = 37.2±12.0, DF) v HC (N=61; 25M; age = 36.9 ± 9.7) | - | p = 0.035 |
| **n-3 PUFAs** | | | | | | | |
|  | (Wang et al., 2018) | LC-MS | serum | ↓ | SCZ (N=115; 51M; age = 29.0 (IQR 25.0-33.2); 88 DF ≥ 1 months, 27 FE) v HC (N=108; 37M; age = 30.0 (IQR 26.0-33.0)) | - | q = 0.015 |
| ﻿all-cis-7,10,13,16,19-DPA(clupanodonic acid)  C22:5 *cis*-7,10,13,16,19 | (Yang et al., 2017) | LC-MS | serum | ↑ | SCZ (N=60; 24M; age = 37.2±12.0, DF) v HC (N=61; 25M; age = 36.9 ± 9.7) | - | p = 0.02 |
| Eicosapentaenoic acid  C 20:5 | (Wang et al., 2018) | LC-MS | serum | ↓ | SCZ (N=115; 51M; age = 29.0 (IQR 25.0-33.2); 88 DF ≥ 1 months, 27 FE) v HC (N=108; 37M; age = 30.0 (IQR 26.0-33.0)) | - | q = 0.001 |
| 12-hydroxy-eicosapentaenoic acid | (Wang et al., 2018) | LC-MS | serum | ↑ | SCZ (N=115; 51M; age = 29.0 (IQR 25.0-33.2); 88 DF ≥ 1 months, 27 FE) v HC (N=108; 37M; age = 30.0 (IQR 26.0-33.0)) | - | q = 0.045 |
| 12-hydroperoxy-eicosatetraenoic acid(C20:4) | (Wang et al., 2018) | LC-MS | serum | ↓ | SCZ (N=115; 51M; age = 29.0 (IQR 25.0-33.2); 88 DF ≥ 1 months, 27 FE) v HC (N=108; 37M; age = 30.0 (IQR 26.0-33.0)) | - | q = 0.03 |
| 12-hydroxy-eicosatetraenoic acid | (Wang et al., 2018) | LC-MS | serum | ↓ | SCZ (N=115; 51M; age = 29.0 (IQR 25.0-33.2); 88 DF ≥ 1 months, 27 FE) v HC (N=108; 37M; age = 30.0 (IQR 26.0-33.0)) | - | q = 0.007 |
| 15-hydroxy-eicosatetraenoic acid | (Wang et al., 2018) | LC-MS | serum | ↑ | SCZ (N=115; 51M; age = 29.0 (IQR 25.0-33.2); 88 DF ≥ 1 months, 27 FE) v HC (N=108; 37M; age = 30.0 (IQR 26.0-33.0)) | - | q = 0.025 |
| 8-hydroxy-eicosatetraenoic acid | (Wang et al., 2018) | LC-MS | serum | ↑ | SCZ (N=115; 51M; age = 29.0 (IQR 25.0-33.2); 88 DF ≥ 1 months, 27 FE) v HC (N=108; 37M; age = 30.0 (IQR 26.0-33.0)) | - | q = 0.023 |
| 11-hydroxy-eicosatetraenoic acid | (Wang et al., 2018) | LC-MS | serum | ↓ | SCZ (N=115; 51M; age = 29.0 (IQR 25.0-33.2); 88 DF ≥ 1 months, 27 FE) v HC (N=108; 37M; age = 30.0 (IQR 26.0-33.0)) | - | q = 0.004 |
| 13-hydroxyl-docosahexaenoic acid | (Wang et al., 2018) | LC-MS | serum | ↓ | SCZ (N=115; 51M; age = 29.0 (IQR 25.0-33.2); 88 DF ≥ 1 months, 27 FE) v HC (N=108; 37M; age = 30.0 (IQR 26.0-33.0)) | - | q = 0.02 |
| 11,12- di-hydroxy-eicosatrienoic acid  C 20:3 | (Wang et al., 2018) | LC-MS | serum | ↑ | SCZ (N=115; 51M; age = 29.0 (IQR 25.0-33.2); 88 DF ≥ 1 months, 27 FE) v HC (N=108; 37M; age = 30.0 (IQR 26.0-33.0)) | - | q = 0.02 |
| 14,15- di-hydroxy-eicosatrienoic acid | (Wang et al., 2018) | LC-MS | serum | ↑ | SCZ (N=115; 51M; age = 29.0 (IQR 25.0-33.2); 88 DF ≥ 1 months, 27 FE) v HC (N=108; 37M; age = 30.0 (IQR 26.0-33.0)) | - | q = 0.0056 |
| **﻿OCFAs** | | | | | | | |
| Cis-10-Heptadecenoic acid  ﻿C 17:1 *cis*-10 | (Yang et al., 2017) | LC-MS | serum | ↑ | SCZ (N=60; 24M; age = 37.2±12.0, DF) v HC (N=61; 25M; age = 36.9 ± 9.7) | - | p = 0.012 |
| 10-cis-Nonadecenoic acid  ﻿C 19:1 *cis*-10 | (Yang et al., 2017) | LC-MS | serum | ↑ | SCZ (N=60; 24M; age = 37.2±12.0, DF) v HC (N=61; 25M; age = 36.9 ± 9.7) | - | p = 0.087 |
| **﻿Fatty acids metabolism compounds** | | | | | | | |
| α-hydroxybutyrate | (Yang et al., 2013) | GC-TOFMS, ^1^H NMR | serum | ↑ | SCZ (N=62; 25M; age = 36.9 ± 11.9; DF ≥ 2 weeks, some FE) v HC (N=62; 25M; age = 36.9 ± 9.3) | - | p <0.001, q <0.02 |
|  | (Yang et al., 2013) | GC-TOFMS, ^1^H NMR | urine | ↑ | SCZ (N=51; DF ≥ 2 weeks, some FE) v HC (N=51) | - | p <0.007; q <0.025 |
|  | (Kageyama et al., 2017) | CE-TOFMS | plasma | ↑ | SCZ (N=17; 8M; age = 33.6 ± 15.7; DF ≥2 wks) v HC (N=19; 10M; age = 36.1 ± 12.9) | - | p = 0.043 |
| β-hydroxybutyrate | (Cai et al., 2012) | ^1^H NMR | plasma | ↓ | FE DN SCZ (N=11; 6M; age = 27.6 ± 9.5) v HC (N=11; 6M; age = 27.6 ± 9.5) | - | p= 0.011 (ns after Bonferroni correction) |
|  | (Yang et al., 2013) | GC-TOFMS, ^1^H NMR | serum | ↑ | SCZ (N=62; 25M; age = 36.9 ± 11.9; DF ≥ 2 weeks, some FE) v HC (N=62; 25M; age = 36.9 ± 9.3) | - | p <0.001, q <0.02 |
|  | (Yang et al., 2013) | GC-TOFMS, ^1^H NMR | urine | ↑ | SCZ (N=51; DF ≥ 2 weeks, some FE) v HC (N=51) | - | p <0.007; q <0.025 |
|  | (Fukushima et al., 2014) | LC-MS | serum | ↓ | med SCZ (N=25; 11M; age = 28.2 ± 4.4) v HC (N=27; 12M; age = 26.5 ± 5.6) | + | p <0.005 (ns after Bonferroni) |
| Suberic acid | (Yang et al., 2013) | GC-TOFMS, ^1^H NMR | urine | ↑ | SCZ (N=51; DF ≥ 2 weeks, some FE) v HC (N=51) | - | p <0.007; q <0.025 |
| 4-Pentenoic acid | (Yang et al., 2013) | GC-TOFMS, ^1^H NMR | urine | ↑ | SCZ (N=51; DF ≥ 2 weeks, some FE) v HC (N=51) | - | p <0.007; q <0.025 |
| **Steroids & derivatives** | | | | | | | |
| DHEAS | (Bicikova et al., 2013) | GC-MS | serum | ↑ | female FE DN SCZ (N=8; age (median) = 35) v HC (N=25; age (median) = 35) | - | p <0.05 |
| Progesterone | (Bicikova et al., 2013) | GC-MS | serum | ↑ | male FE DN SCZ (N=13; age (median) = 31) v male HC (N=22; age (median) = 35) | - | p <0.001 |
|  | (Bicikova et al., 2013) | GC-MS | serum | ↑ | female FE DN SCZ (N=8; age (median) = 35) v HC (N=25; age (median) = 35) | - | p <0.001 |
| 5α-dihydroprogesterone | (Bicikova et al., 2013) | GC-MS | serum | ↑ | female FE DN SCZ (N=8; age (median) = 35) v HC (N=25; age (median) = 35) | - | p <0.001 |
|  | (Bicikova et al., 2013) | GC-MS | serum | ↑ | female FE DN SCZ (N=8; age (median) = 35) v HC (N=25; age (median) = 35) | - | p <0.001 |
| 20α-Dihydroprogesterone | (Bicikova et al., 2013) | GC-MS | serum | ↓ | female FE DN SCZ (N=8; age (median) = 35) v HC (N=25; age (median) = 35) | - | p <0.001 |
| 5α, 20α-tetrahydroprogesterone | (Bicikova et al., 2013) | GC-MS | serum | ↑ | female FE DN SCZ (N=8; age (median) = 35) v HC (N=25; age (median) = 35) | - | p <0.001 |
| Pregnanolone | (Bicikova et al., 2013) | GC-MS | serum | ↓ | female FE DN SCZ (N=8; age (median) = 35) v HC (N=25; age (median) = 35) | - | p <0.001 |
| Conjugated pregnanolone | (Bicikova et al., 2013) | GC-MS | serum | ↑ | male FE DN SCZ (N=13; age (median) = 31) v male HC (N=22; age (median) = 35) | - | p <0.01 |
| Isopregnanolone | (Bicikova et al., 2013) | GC-MS | serum | ↓ | female FE DN SCZ (N=8; age (median) = 35) v HC (N=25; age (median) = 35) | - | p <0.01 |
| Conjugated Isopregnanolone | (Bicikova et al., 2013) | GC-MS | serum | ↑ | male FE DN SCZ (N=13; age (median) = 31) v male HC (N=22; age (median) = 35) | - | p <0.01 |
|  | (Bicikova et al., 2013) | GC-MS | serum | ↑ | female FE DN SCZ (N=8; age (median) = 35) v HC (N=25; age (median) = 35) | - | p <0.001 |
| Conjugated epipregnanolone | (Bicikova et al., 2013) | GC-MS | serum | ↑ | male FE DN SCZ (N=13; age (median) = 31) v male HC (N=22; age (median) = 35) | - | p <0.001 |
| Pregnanediol | (Cai et al., 2012) | ^1^H NMR | urine | ↑ | FE DN SCZ (N=11; 6M; age = 27.6 ± 9.5) v HC (N=11; 6M; age = 27.6 ± 9.5) | - | p = 0.033 (ns after Bonferoni correction) |
| Epietiocholanolone | (Bicikova et al., 2013) | GC-MS | serum | ↓ | female FE DN SCZ (N=8; age (median) = 35) v HC (N=25; age (median) = 35) | - | p <0.001 |
| Conjugated epietiocholanolone | (Bicikova et al., 2013) | GC-MS | serum | ↓ | male FE DN SCZ (N=13; age (median) = 31) v male HC (N=22; age (median) = 35) | - | p <0.01 |
| Etiocholanolone | (Bicikova et al., 2013) | GC-MS | serum | ↓ | female FE DN SCZ (N=8; age (median) = 35) v HC (N=25; age (median) = 35) | - | p <0.001 |
|  | (Bicikova et al., 2013) | GC-MS | serum | ↓ | male FE DN SCZ (N=13; age (median) = 31) v male HC (N=22; age (median) = 35) | - | p <0.001 |
| Pregnenolone | (Bicikova et al., 2013) | GC-MS | serum | ↓ | female FE DN SCZ (N=8; age (median) = 35) v HC (N=25; age (median) = 35) | - | p <0.01 |
|  | (Bicikova et al., 2013) | GC-MS | serum | ↓ | male FE DN SCZ (N=13; age (median) = 31) v male HC (N=22; age (median) = 35) | - | p <0.001 |
| 20α-dihydropregnenolone | (Bicikova et al., 2013) | GC-MS | serum | ↑ | female FE DN SCZ (N=8; age (median) = 35) v HC (N=25; age (median) = 35) | - | p <0.05 |
| Conjuagted pregnenolone sulfate | (Bicikova et al., 2013) | GC-MS | serum | ↑ | male FE DN SCZ (N=13; age (median) = 31) v male HC (N=22; age (median) = 35) | - | p <0.01 |
| Conjugated 5α-Pregnane-3β, 20α-diol | (Bicikova et al., 2013) | GC-MS | serum | ↓ | male FE DN SCZ (N=13; age (median) = 31) v male HC (N=22; age (median) = 35) | - | p <0.001 |
| Androsterone | (Bicikova et al., 2013) | GC-MS | serum | ↓ | male FE DN SCZ (N=13; age (median) = 31) v male HC (N=22; age (median) = 35) | - | p <0.05 |
| Epiandrosterone | (Bicikova et al., 2013) | GC-MS | serum | ↓ | male FE DN SCZ (N=13; age (median) = 31) v male HC (N=22; age (median) = 35) | - | p <0.05 |
| Androstenediol | (Bicikova et al., 2013) | GC-MS | serum | ↓ | male FE DN SCZ (N=13; age (median) = 31) v male HC (N=22; age (median) = 35) | - | p <0.05 |
| Conjugated androstenediol | (Bicikova et al., 2013) | GC-MS | serum | ↑ | female FE DN SCZ (N=8; age (median) = 35) v HC (N=25; age (median) = 35) | - | p <0.01 |
| Androstenedione | (Bicikova et al., 2013) | GC-MS | serum | ↑ | male FE DN SCZ (N=13; age (median) = 31) v male HC (N=22; age (median) = 35) | - | p <0.01 |
| Conjugated 5α-Androstane-3α, 17β-diol | (Bicikova et al., 2013) | GC-MS | serum | ↑ | female FE DN SCZ (N=8; age (median) = 35) v HC (N=25; age (median) = 35) | - | p <0.001 |
| Conjugated 5α-androstane-3β, 17β-diol | (Bicikova et al., 2013) | GC-MS | serum | ↑ | female FE DN SCZ (N=8; age (median) = 35) v HC (N=25; age (median) = 35) | - | p <0.01 |
| Cortisol | (Bicikova et al., 2013) | GC-MS | serum | ↑ | male FE DN SCZ (N=13; age (median) = 31) v male HC (N=22; age (median) = 35) | - | p <0.01 |
| Testosterone | (Bicikova et al., 2013) | GC-MS | serum | ↑ | female FE DN SCZ (N=8; age (median) = 35) v HC (N=25; age (median) = 35) | - | p <0.001 |
| **Other lipids & lipid-like molecules** | | | | | | | |
| CE (16:1, 18:1, 18:3, 20:3, 20:4, 22:6) | (Yan et al., 2018) | LC-MS | plasma | ↑ | SCZ (N=20, M=11, DN); HC (N=29, M=18) | - | P <0.05 |
| LPE (16:0, 18:1) | (Yan et al., 2018) | LC-MS | plasma | ↓ | SCZ (N=20, M=11, DN); HC (N=29, M=18) | - | P <0.05 |
| Plasmenyl-PE (16:0/18:1, 16:0/18:2, 18:0/18:1, 18:0/18:2, 18:0/18:3, 16:0/20:4, 20:0/18:2, 18:0/20:3, 18:0/20:4, 16:0/22:5, 16:0/22:6, 18:0/22:4, 18:0/22:6) | (Yan et al., 2018) | LC-MS | plasma | ↓ | SCZ (N=20, M=11, DN); HC (N=29, M=18) | - | P <0.05 |
| PC (16:0/18:2) | (Cai et al., 2012) | ^1^H NMR | plasma | ↓ | FE DN SCZ (N=11; 6M; age = 27.6 ± 9.5) v HC (N=11; 6M; age = 27.6 ± 9.5) | - | p = 0.038 (ns after Bonferroni correction) |
| PC (18:2/18:2, 18:2/18:3) | (Yan et al., 2018) | LC-MS | plasma | ↓ | SCZ (N=20, M=11, DN); HC (N=29, M=18) | - | P <0.05 |
| PC aa C30:0, C32:1, C32:2, C34:2, C34:3, C34:4, C36:1, C36:2, C36:3, C36:6, C38.3 | (Leppik et al., 2020) | LC-MS | serum | ↓ | DN FEP (N=53; 32M; age = 26.2 ± 6.0) v HC (N=37; 16M; age = 24.8 ± 5.3) | - | P < 5.0 × 10^-4^ |
| PC ae C34:2, C36:2, C36:3, C40:2, C40:4 | (Leppik et al., 2020) | LC-MS | serum | ↓ | DN FEP (N=53; 32M; age = 26.2 ± 6.0) v HC (N=37; 16M; age = 24.8 ± 5.3) | - | P < 5.0 × 10^-4^ |
| PC ae C38:6 | (He et al., 2012) | FIA-MS | plasma | ↓ | SCZ (N=52 DF (29M; age = 39.3 ± 11.2), N=213 med (132 M; age = 36.9 ± 11.7)) v HC (N=216; 112M; age = 38.9 ± 10.6) | + | p = 0.03 |
| LPC (16:0); LPC(18:0); LPC(18:1); LPC(18:2) | (Cai et al., 2012) | ^1^H NMR | plasma | ↑ | FE DN SCZ (N=11; 6M; age = 27.6 ± 9.5) v HC (N=11; 6M; age = 27.6 ± 9.5) | - | p <0.05 |
| LPC (16:0); LPC(18:0); LPC(20:3) | (Orešič et al., 2012) | LC-MS/CE-TOFMS | serum | ↓ | SCZ twins (N=19; 15 med; 6M; 7 MZ; age = 51 ± 10) v unaffected co-twins (N=19; matched gender); SCZ v HC twins (N=34; 10M; 20 MZ; age = 53.4) | + | p<0.05 |
| LPC (14:0, 18:0, 18:2, 20:0, 20:2, 22:0,22:6) | (Yan et al., 2018) | LC-MS | plasma | ↓ | SCZ (N=20, M=11, DN); HC (N=29, M=18) | - | p<0.05 |
| LPC (20:4) | (Leppik et al., 2020) | LC-MS | serum | ↑ | DN FEP (N=53; 32M; age = 26.2 ± 6.0) v HC (N=37; 16M; age = 24.8 ± 5.3) | - | p = 2.0 × 10^-4^ |
| LPC (22:4) | (Yan et al., 2018) | LC-MS | plasma | ↑ | SCZ (N=20, M=11, DN); HC (N=29, M=18) | - | P = 0.0025 |
| Plasmenyl-PC (14:0/2:0, 14:0/20:2, 16:0/18:2, 16:0/20:4, 18:0/20:4, 20:2/18:5) | (Yan et al., 2018) | LC-MS | plasma | ↓ | SCZ (N=20, M=11, DN); HC (N=29, M=18) | - | P < 0.05 |
| TG (52:2) | (Orešič et al., 2012) | LC-MS/CE-TOFMS | serum | ↑ | SCZ twins (N=19; 15 med; 6M; 7 MZ; age = 51 ± 10) v unaffected co-twins (N=19; matched gender); SCZ v HC twins (N=34; 10M; 20 MZ; age = 53.4) | + | p<0.05 |
| TG (16:0/16:0/18:1, 16:0/18:1/18:1, 16:0/20:1/20:3, 16:0/18:1/22:4, 18:1/18:1/20:4, 18:1/18:2/20:4, 18:2/18:2/22:6, 18:1/20:4/20:4) | (Yan et al., 2018) | LC-MS | plasma | ↑ | SCZ (N=20, M=11, DN); HC (N=29, M=18) | - | P < 0.05 |
| SM | (Tessier et al., 2016) | LC-MS | RBCs | ↓ | SCZ (N=74; 48M; age = 43.8 ± 9.3) v HC (N=40; 24M; age = 42.6 ± 13.2) | + | p = 3.0 × 10^-4^ |
| SM (d18:1/18:0) | (Orešič et al., 2012) | LC-MS/CE-TOFMS | serum | ↑ | SCZ twins (N=19; 15 med; 6M; 7 MZ; age = 51 ± 10) v unaffected co-twins (N=19; matched gender); SCZ v HC twins (N=34; 10M; 20 MZ; age = 53.4) | + | p<0.05 |
| SM (d14:2/26:2) | (Yan et al., 2018) | LC-MS | plasma | ↑ | SCZ (N=20, M=11, DN); HC (N=29, M=18) | - | p= 2.5 × 10^-6^ |
| SM (C20:2) | (Leppik et al., 2020) | LC-MS | serum | ↓ | DN FEP (N=53; 32M; age = 26.2 ± 6.0) v HC (N=37; 16M; age = 24.8 ± 5.3) | - | p = 9.0 × 10^-4^ |
| Glycerol 3-phosphate | (Liu et al., 2015) | GC-MS | PBMCs | ↓ | Training set: FE DN SCZ (N=35; 14M; age = 32.5 ± 14.1) v HC (N=35; 18M; age = 36.5 ± 6.0)  Test set: SCZ (N=20; 9M; 6 med; age = 28.5 ± 2.1) v HC (N=20; 10M; age = 30.2 ± 1.7) | -, + | P <0.001 training set;  p = 0.001 test set |
| Perillic acid | (Koike et al., 2014) | CE-TOFMS | plasma | ↓ | 1^st^ set: FE SCZ (N= 18; 13M; most med, 2 DN; 4 disorganised, 7 paranoid, 3 SFD, 2 delusional disorder, 2 PD NOS; age = 23.2 ± 5.4) v HC (N=14; 11M; age = 25.7 ± 6.1)  2^nd^ set: FE SCZ (N=12; 4 disorganised, 3 paranoid, 3 SFD, 2 PD NOS; 2 DN; age = 24.6 ± 7.1) v HC (N=24 HC; 10M; age = 26.1 ± 2.6) | +, + | p = 0.018 (1st set)  p = 0.0040 (2nd set) |
| Malate | (Yang et al., 2013) | GC-TOFMS, ^1^H NMR | serum | ↑ | SCZ (N=62; 25M; age = 36.9 ± 11.9; DF ≥ 2 weeks, some FE) v HC (N=62; 25M; age = 36.9 ± 9.3) | - | p =2.21 x 10^-8^, q =1.82 x 10^-3^ |
| **Clusters** | | | | | | | |
| LC4: Short chain saturated TGs | (Orešič et al., 2011) | GC-TOFMS | serum | ↑ | SCZ (N=45; 19M; 34 med; age = 53.7 ± 12.9) v HC (N=45; 19M; age = 53.7 ± 12.9) | + | p = 0.0003 |
| LC5  Mainly unidentified, includes short odd-chain TG | (Orešič et al., 2011) | GC-TOFMS | serum | ↑ | SCZ (N=45; 19M; 34 med; age = 53.7 ± 12.9) v HC (N=45; 19M; age = 53.7 ± 12.9) | + | p = 0.045 |
| LC5 (abundant triglycerides) | (Orešič et al., 2012) | LC-MS/CE-TOFMS | Serum | ↑ | SCZ twins (N=19; 15 med; 6M; 7 MZ; age = 51 ± 10) v unaffected co-twins (N=19; matched gender); SCZ v HC twins (N=34; 10M; 20 MZ; age = 53.4) | + | p = 0.039 (SCZ twins v co-twins); p = ns (SCZ twins v HC & HC v co-twins) |
| LC6  Odd-chain TGs, mainly saturated or monounsaturated | (Orešič et al., 2011) | GC-TOFMS | serum | ↑ | SCZ (N=45; 19M; 34 med; age = 53.7 ± 12.9) v HC (N=45; 19M; age = 53.7 ± 12.9) | + | p = 0.025 |
| LC7  Mainly odd-chain TGs, longer fatty acids than LC5 and LC6 | (Orešič et al., 2011) | GC-TOFMS | serum | ↑ | SCZ (N=45; 19M; 34 med; age = 53.7 ± 12.9) v HC (N=45; 19M; age = 53.7 ± 12.9) | + | p = 0.002 |
| LC8  Medium- and long-chain TGs | (Orešič et al., 2011) | GC-TOFMS | serum | ↑ | SCZ (N=45; 19M; 34 med; age = 53.7 ± 12.9) v HC (N=45; 19M; age = 53.7 ± 12.9) | + | p = 0.003 |
| LC9  Longer-chain, SFA- and MUFA-containing TGs | (Orešič et al., 2011) | GC-TOFMS | serum | ↑ | SCZ (N=45; 19M; 34 med; age = 53.7 ± 12.9) v HC (N=45; 19M; age = 53.7 ± 12.9) | + | p <0.0001 |
| MC2 (ketone bodies, free fatty acids) | (Orešič et al., 2011) | GC-TOFMS | serum | ↓ | SCZ (N=45; 19M; 34 med; age = 53.7 ± 12.9) v HC (N=45; 19M; age = 53.7 ± 12.9)  affective psychosis (N=37; 23M; 8 med; age = 54.7 ± 14.8) v HC (N=37; 23M; age = 54.7 ± 14.9) | + | p = 0.009 (SCZ as predictor) ↓ in affective psychosis (p = 0.006) ↑ with AP use (P = 0.016) |
| lipid (1.58ppm) | (Cai et al., 2012) | ^1^H NMR | Plasma | ↓ | FE DN SCZ (N=11; 6M; age = 27.6 ± 9.5) v HC (N=11; 6M; age = 27.6 ± 9.5) | - | p = 0.009 (ns after Bonferoni correction) |
| UFA (unsaturated fatty acids; 5.26-5.30ppm) | (Cai et al., 2012) | ^1^H NMR | Plasma | ↓ | FE DN SCZ (N=11; 6M; age = 27.6 ± 9.5) v HC (N=11; 6M; age = 27.6 ± 9.5) | - | p = 0.014 ( after Bonferroni correction) |
| Cholesterol | (Al Awam et al., 2015) | FTIR, GC-MS | serum | ↓ | med SCZ (N=26; 20M; age = 37.3 ± 12.4) v HC (N=26; 20M; age = 37.0 ± 10.7) | + | p <0.001 |
|  | (Xuan et al., 2011) | GC-MS | serum | ↑ | DF SCZ (N=18 ; 10M; age = 38 ± 15); HC (N=18 ; 10M; age = 41 ± 19) | - | p = 0.029 |
| VLDL (very low density lipoproteins; 0.88-0.92 ppm; 1.28-1.32 ppm) | (Tsang et al., 2006) | ^1^H NMR | plasma | ↑ | Female med SCZ affected MZ twins (N=8; age = 33.9 ± 6.4) v unaffected co-twins (N=8)  Female med SCZ affected MZ twins v HC twins (N=10; age = 29.3 ± 6.4) | + | p <0.05 |
| VLDL (very low density lipoproteins; 1.26ppm) | (Cai et al., 2012) | ^1^H NMR | Plasma | ↓ | FE DN SCZ (N=11; 6M; age = 27.6 ± 9.5) v HC (N=11; 6M; age = 27.6 ± 9.5) | - | p = 0.020 (ns after Bonferoni correction) |
| VLDL/LDL (low and very low density lipoproteins; 1.30-1.34ppm) | (Cai et al., 2012) | ^1^H NMR | Plasma | ↓ | FE DN SCZ (N=11; 6M; age = 27.6 ± 9.5) v HC (N=11; 6M; age = 27.6 ± 9.5) | - | p = 0.009 (ns after Bonferoni correction) |
| LDL (low density lipoproteins; 0.84-0.88 ppm; 1.24-1.28 ppm) | (Tsang et al., 2006) | ^1^H NMR | plasma | ↑ | Female med SCZ affected MZ twins (N=8; age = 33.9 ± 6.4) v unaffected co-twins (N=8)  Female med SCZ affected MZ twins v HC twins (N=10; age = 29.3 ± 6.4) | + | p <0.05 |
| LDL (low density lipoproteins; 0.82-0.86ppm) | (Cai et al., 2012) | ^1^H NMR | Plasma | ↓ | FE DN SCZ (N=11; 6M; age = 27.6 ± 9.5) v HC (N=11; 6M; age = 27.6 ± 9.5) | - | p = 0.039 (ns after Bonferoni correction) |
| HDL (high density lipoproteins; 1.18-1.22ppm) | (Cai et al., 2012) | ^1^H NMR | Plasma | ↓ | FE DN SCZ (N=11; 6M; age = 27.6 ± 9.5) v HC (N=11; 6M; age = 27.6 ± 9.5) | - | p = 0.015 (ns after Bonferoni correction) |

DHEAS = dehydroepiandrosterone sulfate; PC = phosphatidylcholine; PE = phosphatidylethaolamine; LPC = lysophosphatidylcholine; TG = triglycerides; n3/6/7/9 = omega 3/6/7/9; UFA = unsaturated fatty acids; MUFA = monosaturated fatty acids; PUFA = poly-unsaturated fatty acids; LDL = low density lipoprotein; VLDL = very low density lipoprotein; HDL = high density lipoprotein; GC = gas chromatography; LC =liquid chromatography; MS = mass spectrometry; ^1^H NMR = proton nuclear magnetic resonance spectroscopy; TOFMS = time-of-flight MS; CE = capillary electrophoresis; RBCs = red blood cells; PBMCs = peripheral blood mononuclear cells; SCZ = schizophrenia subjects; HC = healthy control subjects; med = taking medication; FE = first episode; RE = recurrent episode; DN = drug naïve; DF = drug-free; SZA = schizoaffective disorder; M = male; F = female

**Table s5: Organic acids found at abnormal levels in patients with schizophrenia**

| **Metabolite** | **Reference** | **Platform** | **biofluid** | **difference (SCZ v control)** | **subjects** | **Medication** | **P value** | |
| --- | --- | --- | --- | --- | --- | --- | --- | --- |
| **Amino acids** | | | | | | | |  |
| 1-oxoproline | (Al Awam et al., 2015) | FTIR, GC-MS | serum | ↓ | med SCZ (N=26; 20M; age = 37.3 ± 12.4) v HC (N=26; 20M; age = 37.0 ± 10.7) | + | p <0.01 | |
| 2-Aminobutyric acid | (Yang et al., 2013) | GC-TOFMS, ^1^H NMR | serum | ↑ | SCZ (N=62; 25M; age = 36.9 ± 11.9; DF ≥ 2 weeks, some FE) v HC (N=62; 25M; age = 36.9 ± 9.3) | - | p <0.025, q <0.035 | |
|  | (Yang et al., 2013) | GC-TOFMS, ^1^H NMR | urine | ↑ | SCZ (N=51; DF ≥ 2 weeks, some FE) v HC (N=51) | - | p <0.04; q <0.045 | |
| 5-Oxoproline (pyroglutamic acid) | (Yang et al., 2013) | GC-TOFMS, ^1^H NMR | serum | ↑ | SCZ (N=62; 25M; age = 36.9 ± 11.9; DF ≥ 2 weeks, some FE) v HC (N=62; 25M; age = 36.9 ± 9.3) | - | p <0.025, q <0.035 | |
|  | (Yang et al., 2013) | GC-TOFMS, ^1^H NMR | urine | ↑ | SCZ (N=51; DF ≥ 2 weeks, some FE) v HC (N=51) | - | p <0.04; q <0.045 | |
|  | (Yoshikawa et al., 2018) | CE-TOFMS | plasma | ↑ | SCZ (N=5; 2M; age = 50.2 ± 14.33) v HC (N=5; 0M; age = 39.0 ± 2.28) | + ? | p <0.05 | |
| α-Aminoadipic acid (AAA) | (Yang et al., 2013) | GC-TOFMS, ^1^H NMR | urine | ↓ | SCZ (N=51; DF ≥ 2 weeks, some FE) v HC (N=51) | - | p <0.007, q <0.035 | |
|  | (Parksepp et al., 2020) | FIA-MS, LC-MS | serum | ↓ | DN FEP (N=52; 31M; age = 27.0 ± 6.1) v HC (N=37; 16M; age = 24.9 ± 5.3) | - | p<0.0001 | |
| AAA/KYN | (Parksepp et al., 2020) | FIA-MS, LC-MS | serum | ↓ | FEP (N=37; 23M; age = 32.0 ± 5.9; medicated for 5.1 yrs) v HC (N=37; 16M; age = 24.9 ± 5.3) | + | p<0.0001 | |
| Alanine | (Cai et al., 2012) | ^1^H NMR | plasma | ↑ | FE DN SCZ (N=11; 6M; age = 27.6 ± 9.5) v HC (N=11; 6M; age = 27.6 ± 9.5) | - | p = 0.047 (ns after Bonferoni correction) | |
|  | Ours | CE-TOFMS/ LC-TOFMS | serum | ↑ | SCZ (N=20; 10M; 43.4 ± 8.8) v HC (N=20; 10M; age = 41.8 ± 12.2) | + | p = 7.3 × 10^-7^ | |
| Arginine | (He et al., 2012) | FIA-MS | plasma | ↓ | SCZ (N=52 DF (29M; age = 39.3 ± 11.2), N=213 med (132 M; age = 36.9 ± 11.7)) v HC (N=216; 112M; age = 38.9 ± 10.6) | + | p ≤0.03 | |
|  | (Cao et al., 2018) | LC-MS | plasma | ↓ | SCZ (N = 208 (40DN&FE, 168 DF > 1 month); 135M; age = 37.77 ± 10.85)v HC (N=175; 54M; age = 39.44 ± 9.36) | + | p <0.001 | |
| Aspartate (aspartic acid) | (Xuan et al., 2011) | GC-MS | serum | ↓ | DF SCZ (N=18 ; 10M; age = 38 ± 15); HC (N=18 ; 10M; age = 41 ± 19) | - | p = 0.0021 | |
|  | (Yang et al., 2013) | GC-TOFMS, ^1^H NMR | serum | ↑ | SCZ (N=62; 25M; age = 36.9 ± 11.9; DF ≥ 2 weeks, some FE) v HC (N=62; 25M; age = 36.9 ± 9.3) | - | p <0.025, q <0.035 | |
|  | (Liu et al., 2014) | GC-MS | PBMCs | ↑ | SCZ (N=45; 18M; 19 FE DN, 26 med; age = 33.2 ± 12.9) v HC (N=50; 22M; age = 37.3 ± 8.7) | + | p <0.04 | |
|  | (Yoshikawa et al., 2018) | CE-TOFMS | plasma | ↑ | SCZ (N=5; 2M; age = 50.2 ± 14.33) v HC (N=5; 0M; age = 39.0 ± 2.28) | + ? | p <0.05 | |
|  | (Parksepp et al., 2020) | FIA-MS, LC-MS | serum | ↓ | FEP (N=37; 23M; age = 32.0 ± 5.9; medicated for 5.1 yrs) v HC (N=37; 16M; age = 24.9 ± 5.3) | + | p<0.0001 | |
| Asp/Asn | (Parksepp et al., 2020) | FIA-MS, LC-MS | serum | ↓ | FEP (N=37; 23M; age = 32.0 ± 5.9; medicated for 5.1 yrs) v HC (N=37; 16M; age = 24.9 ± 5.3) | + | p<0.0001 | |
| Aspargine | (Parksepp et al., 2020) | FIA-MS, LC-MS | serum | ↑ | FEP (N=37; 23M; age = 32.0 ± 5.9; medicated for 5.1 yrs) v HC (N=37; 16M; age = 24.9 ± 5.3) | + | p<0.0001 | |
| N-acetylaspartate | (Xuan et al., 2011) | GC-MS | serum | ↓ | DF SCZ (N=18 ; 10M; age = 38 ± 15); HC (N=18 ; 10M; age = 41 ± 19) | - | p = 0.0293 | |
| Betaine | (Koike et al., 2014) | CE-TOFMS | plasma | ↓ | 1^st^ set: FE SCZ (N= 18; 13M; most med, 2 DN; 4 disorganised, 7 paranoid, 3 SFD, 2 delusional disorder, 2 PD NOS; age = 23.2 ± 5.4) v HC (N=14; 11M; age = 25.7 ± 6.1)  2^nd^ set: FE SCZ (N=12; 4 disorganised, 3 paranoid, 3 SFD, 2 PD NOS; 2 DN; age = 24.6 ± 7.1) v HC (N=24 HC; 10M; age = 26.1 ± 2.6) | + | p = 6.8 x 10^-4^ (1st set)  p = 0.029 (2nd set) | |
| Creatine | (Cai et al., 2012) | ^1^H NMR | Urine | ↓ | FE DN SCZ (N=11; 6M; age = 27.6 ± 9.5) v HC (N=11; 6M; age = 27.6 ± 9.5) | - | p = 0.039 (ns after Bonferoni correction) | |
|  | (Koike et al., 2014) | CE-TOFMS | plasma | ↑ | 1^st^ set: FE SCZ (N= 18; 13M; most med, 2 DN; 4 disorganised, 7 paranoid, 3 SFD, 2 delusional disorder, 2 PD NOS; age = 23.2 ± 5.4) v HC (N=14; 11M; age = 25.7 ± 6.1)  2^nd^ set: FE SCZ (N=12; 4 disorganised, 3 paranoid, 3 SFD, 2 PD NOS; 2 DN; age = 24.6 ± 7.1) v HC (N=24 HC; 10M; age = 26.1 ± 2.6) | + | p = 0.018 (1st set)  p=0.031 (2nd set) | |
|  | (Kageyama et al., 2017) | CE-TOFMS | plasma | ↓ | SCZ (N=17; 8M; age = 33.6 ± 15.7; DF ≥2 wks) v HC (N=19; 10M; age = 36.1 ± 12.9) | - | p = 0.016 | |
| Creatinine | (Cai et al., 2012) | ^1^H NMR | urine | ↓ | FE DN SCZ (N=11; 6M; age = 27.6 ± 9.5) v HC (N=11; 6M; age = 27.6 ± 9.5) | - | p = 0.039 (UPLC-MS/MS)  p = 0.014 (^1^H NMR) (ns after Bonferoni correction) | |
|  | (Liu et al., 2014) | GC-MS | PBMCs | ↓ | SCZ (N=45; 18M; 19 FE DN, 26 med; age = 33.2 ± 12.9) v HC (N=50; 22M; age = 37.3 ± 8.7) | + | p <0.02 | |
| Cystine | (Yang et al., 2013) | GC-TOFMS, ^1^H NMR | serum | ↓ | SCZ (N=62; 25M; age = 36.9 ± 11.9; DF ≥ 2 weeks, some FE) v HC (N=62; 25M; age = 36.9 ± 9.3) | - | p = 1.06 x 10^-6^, q = 0.00424 | |
|  | (Yang et al., 2013) | GC-TOFMS, ^1^H NMR | urine | ↑ | SCZ (N=51; DF ≥ 2 weeks, some FE) v HC (N=51) | - | p <0.04; q <0.045 | |
| Cysteine | (Cao et al., 2018) | LC-MS | plasma | ↑ | SCZ (N = 208 (40DN&FE, 168 DF > 1 month); 135M; age = 37.77 ± 10.85)　v HC (N=175; 54M; age = 39.44 ± 9.36) | + | p = 0.006 | |
| Methylcysteine | (Cao et al., 2018) | LC-MS | plasma | ↓ | SCZ (N = 208 (40DN&FE, 168 DF > 1 month); 135M; age = 37.77 ± 10.85)　v HC (N=175; 54M; age = 39.44 ± 9.36) | + | P = 0.01 | |
| γ-glutamylcysteine | (Fukushima et al., 2014) | LC-MS | serum | ↓ | med SCZ (N=25; 11M; age = 28.2 ± 4.4) v HC (N=27; 12M; age = 26.5 ± 5.6) | + | p = 1.75 x 10^-6^ (also sig after Bonferroni correction) | |
| Serine | (Yang et al., 2013) | GC-TOFMS, ^1^H NMR | serum | ↑ | SCZ (N=62; 25M; age = 36.9 ± 11.9; DF ≥ 2 weeks, some FE) v HC (N=62; 25M; age = 36.9 ± 9.3) | - | p <0.025, q <0.035 | |
|  | Ours | CE-TOFMS/ LC-TOFMS | serum | ↑ | SCZ (N=20; 10M; 43.4 ± 8.8) v HC (N=20; 10M; age = 41.8 ± 12.2) | + | p = 4.7 × 10^-6^ | |
| D-Serine | (Fukushima et al., 2014) | LC-MS | serum | ↓ | med SCZ (N=25; 11M; age = 28.2 ± 4.4) v HC (N=27; 12M; age = 26.5 ± 5.6) | + | p = 0.0016 (ns after Bonferroni correction) | |
| γ-Aminobutyric acid (GABA) | (Cao et al., 2018) | LC-MS | plasma | ↑ | SCZ (N = 208 (40DN&FE, 168 DF > 1 month); 135M; age = 37.77 ± 10.85)v HC (N=175; 54M; age = 39.44 ± 9.36) | + | p < 0.001 | |
| Glutamate (glutamic acid) | (Yang et al., 2013) | GC-TOFMS, ^1^H NMR | serum | ↑ | SCZ (N=62; 25M; age = 36.9 ± 11.9; DF ≥ 2 weeks, some FE) v HC (N=62; 25M; age = 36.9 ± 9.3) | - | p <0.025, q <0.035 | |
|  | (Yang et al., 2013) | GC-TOFMS, ^1^H NMR | urine | ↑ | SCZ (N=51; DF ≥ 2 weeks, some FE) v HC (N=51) | - | p <0.04; q <0.045 | |
|  | (Fukushima et al., 2014) | LC-MS | serum | ↑ | med SCZ (N=25; 11M; age = 28.2 ± 4.4) v HC (N=27; 12M; age = 26.5 ± 5.6) | + | p = 0.0145 (ns after Bonferroni correction) | |
|  | (Koike et al., 2014) | CE-TOFMS | plasma | ↑ | 1^st^ set: FE SCZ (N= 18; 13M; most med, 2 DN; 4 disorganised, 7 paranoid, 3 SFD, 2 delusional disorder, 2 PD NOS; age = 23.2 ± 5.4) v HC (N=14; 11M; age = 25.7 ± 6.1)  2^nd^ set: FE SCZ (N=12; 4 disorganised, 3 paranoid, 3 SFD, 2 PD NOS; 2 DN; age = 24.6 ± 7.1) v HC (N=24 HC; 10M; age = 26.1 ± 2.6) | + | p = 0.049 (1st set)  p = 0.064 (ns, 2nd set) | |
|  | (Yoshikawa et al., 2018) | CE-TOFMS | plasma | ↑ | SCZ (N=5; 2M; age = 50.2 ± 14.33) v HC (N=5; 0M; age = 39.0 ± 2.28) | + ? | P < 0.05 | |
|  | (Parksepp et al., 2020) | FIA-MS, LC-MS | serum | ↓ | FEP (N=37; 23M; age = 32.0 ± 5.9; medicated for 5.1 yrs) v HC (N=37; 16M; age = 24.9 ± 5.3) | + | p<0.0001 | |
|  | Ours | CE-TOFMS/ LC-TOFMS | serum | ↑ | SCZ (N=20; 10M; 43.4 ± 8.8) v HC (N=20; 10M; age = 41.8 ± 12.2) | + | p = 3.0 × 10^-6^ | |
| Glu/Gln | (Parksepp et al., 2020) | FIA-MS, LC-MS | serum | ↓ | FEP (N=37; 23M; age = 32.0 ± 5.9; medicated for 5.1 yrs) v HC (N=37; 16M; age = 24.9 ± 5.3) | + | p<0.0001 | |
| Pyroglutamic acid | (Liu et al., 2014) | GC-MS | PBMCs | ↓ | SCZ (N=45; 18M; 19 FE DN, 26 med; age = 33.2 ± 12.9) v HC (N=50; 22M; age = 37.3 ± 8.7) | + | p <0.02 | |
| Glutamine | (He et al., 2012) | FIA-MS | plasma | ↓ | SCZ (N=52 DF (29M; age = 39.3 ± 11.2), N=213 med (132 M; age = 36.9 ± 11.7)) v HC (N=216; 112M; age = 38.9 ± 10.6) | + | p = 0.006 | |
|  | (Cao et al., 2018) | LC-MS | plasma | ↑ | SCZ (N = 208 (40DN&FE, 168 DF > 1 month); 135M; age = 37.77 ± 10.85)v HC (N=175; 54M; age = 39.44 ± 9.36) | + | p < 0.001 | |
|  | (Parksepp et al., 2020) | FIA-MS, LC-MS | serum | ↓ | FEP (N=37; 23M; age = 32.0 ± 5.9; medicated for 5.1 yrs) v HC (N=37; 16M; age = 24.9 ± 5.3) | + | p<0.0001 | |
| N-acetyl-glutamine | (Huang et al., 2019) | LC-MS | serum | ↑ | FE SCZ (N=30; 13M; age = 26.8 ± 7.4) v HC (N=60; 26M; age = 27.1 ± 3.4) | - | p = 0.002(crude adjusted) | |
| Glutathione (GSH) | (Fukushima et al., 2014) | LC-MS | serum | ↓ | med SCZ (N=25; 11M; age = 28.2 ± 4.4) v HC (N=27; 12M; age = 26.5 ± 5.6) | + | p <0.05 (ns after Bonferroni correction) | |
| Glutathione (GSSG) | (Ballesteros et al., 2013) |  | blood | ↑ | med SCZ (N=29; 20M; age = 41.1 ± 13.8) v HC (N=25; 11M; age = 38.8 ± 13.7) | + | p = 0.005 (levels of GSSG)  p = 0.023 (% GSSG) | |
| Glycine | (Xuan et al., 2011) | GC-MS | serum | ↓ | DF SCZ (N=18 ; 10M; age = 38 ± 15); HC (N=18 ; 10M; age = 41 ± 19) | - | p = 0.0232 | |
|  | (Cai et al., 2012) | ^1^H NMR | plasma; urine | ↑ | FE DN SCZ (N=11; 6M; age = 27.6 ± 9.5) v HC (N=11; 6M; age = 27.6 ± 9.5) | - | p <0.015 (ns after Bonferoni correction) | |
| Glycocyamine | (Yang et al., 2013) | GC-TOFMS, ^1^H NMR | urine | ↓ | SCZ (N=51; DF ≥ 2 weeks, some FE) v HC (N=51) | - | p <0.007, q <0.035 | |
| Histidine | (He et al., 2012) | FIA-MS | plasma | ↓ | SCZ (N=52 DF (29M; age = 39.3 ± 11.2), N=213 med (132 M; age = 36.9 ± 11.7)) v HC (N=216; 112M; age = 38.9 ± 10.6) | + | p ≤0.03 | |
| Homoserine | (Liu et al., 2014) | GC-MS | PBMCs | ↑ | SCZ (N=45; 18M; 19 FE DN, 26 med; age = 33.2 ± 12.9) v HC (N=50; 22M; age = 37.3 ± 8.7) | + | p <0.04 | |
| N6-acetyl-L-lysine | (Huang et al., 2019) | LC-MS | serum | ↓ | FE SCZ (N=30; 13M; age = 26.8 ± 7.4) v HC (N=60; 26M; age = 27.1 ± 3.4) | - | p = 0.016(crude adjusted) | |
| Methionine | (Parksepp et al., 2020) | FIA-MS, LC-MS | serum | ↑ | FEP (N=37; 23M; age = 32.0 ± 5.9; medicated for 5.1 yrs) v HC (N=37; 16M; age = 24.9 ± 5.3) | + | p<0.0001 | |
| Ornithine | (He et al., 2012) | FIA-MS | plasma | ↑ | SCZ (N=52 DF (29M; age = 39.3 ± 11.2), N=213 med (132 M; age = 36.9 ± 11.7)) v HC (N=216; 112M; age = 38.9 ± 10.6) | + | p = 0.01 | |
|  | (Parksepp et al., 2020) | FIA-MS, LC-MS | serum | ↑ | FEP (N=37; 23M; age = 32.0 ± 5.9; medicated for 5.1 yrs) v HC (N=37; 16M; age = 24.9 ± 5.3) | + | p<0.0009 | |
|  | Ours | CE-TOFMS/ LC-TOFMS | serum | ↑ | SCZ (N=20; 10M; 43.4 ± 8.8) v HC (N=20; 10M; age = 41.8 ± 12.2) | + | p = 1.1 × 10^-5^ | |
| Orn/Arg | (Parksepp et al., 2020) | FIA-MS, LC-MS | serum | ↑ | FEP (N=37; 23M; age = 32.0 ± 5.9; medicated for 5.1 yrs) v HC (N=37; 16M; age = 24.9 ± 5.3) | + | p<0.0001 | |
| L-ornithine | (Cao et al., 2018) | LC-MS | plasma | ↓ | SCZ (N = 208 (40DN&FE, 168 DF > 1 month); 135M; age = 37.77 ± 10.85)v HC (N=175; 54M; age = 39.44 ± 9.36) | + | p < 0.001 | |
| Phenylalanine | (Yang et al., 2013) | GC-TOFMS, ^1^H NMR | serum | ↑ | SCZ (N=62; 25M; age = 36.9 ± 11.9; DF ≥ 2 weeks, some FE) v HC (N=62; 25M; age = 36.9 ± 9.3) | - | p <0.025, q <0.035 | |
| Pipecolinic acid (2-piperidinecarboxylic acid) | (Al Awam et al., 2015) | FTIR, GC-MS | serum | ↑ | med SCZ (N=26; 20M; age = 37.3 ± 12.4) v HC (N=26; 20M; age = 37.0 ± 10.7) | + | p <0.001 | |
|  | (Yang et al., 2013) | GC-TOFMS, ^1^H NMR | urine | ↑ | SCZ (N=51; DF ≥ 2 weeks, some FE) v HC (N=51) | - | p <0.04; q <0.045 | |
| Sarcosine | (Cao et al., 2018) | LC-MS | plasma | ↑ | SCZ (N = 208 (40DN&FE, 168 DF > 1 month); 135M; age = 37.77 ± 10.85)v HC (N=175; 54M; age = 39.44 ± 9.36) | + | p <0.001 | |
| Taurine | (Cai et al., 2012) | ^1^H NMR | Urine | ↓ | FE DN SCZ (N=11; 6M; age = 27.6 ± 9.5) v HC (N=11; 6M; age = 27.6 ± 9.5) | - | p = 0.013 (ns after Bonferoni correction) | |
|  | (Koido et al., 2016) | FIA-MS, LC-MS | serum | ↑ | DN FEP (N=38) v HC (N=37) | - | p < 10^-6^ | |
|  | (Cao et al., 2018) | LC-MS | plasma | ↓ | SCZ (N = 208 (40DN&FE, 168 DF > 1 month); 135M; age = 37.77 ± 10.85)v HC (N=175; 54M; age = 39.44 ± 9.36) | + | p < 0.001 | |
|  | (Parksepp et al., 2020) | FIA-MS, LC-MS | serum | ↑ | FEP (N=37; 23M; age = 32.0 ± 5.9; medicated for 5.1 yrs) v HC (N=37; 16M; age = 24.9 ± 5.3) | + | p<0.0001 | |
| Threonine | (Fukushima et al., 2014) | LC-MS | serum | ↓ | med SCZ (N=25; 11M; age = 28.2 ± 4.4) v HC (N=27; 12M; age = 26.5 ± 5.6) | + | p <0.05 (ns after Bonferroni correction) | |
| Threonine | (Cao et al., 2018) | LC-MS | plasma | ↓ | SCZ (N = 208 (40DN&FE, 168 DF > 1 month); 135M; age = 37.77 ± 10.85)v HC (N=175; 54M; age = 39.44 ± 9.36) | + | p <0.001 | |
| Tyrosine | (Fukushima et al., 2014) | LC-MS | serum | ↓ | med SCZ (N=25; 11M; age = 28.2 ± 4.4) v HC (N=27; 12M; age = 26.5 ± 5.6) | + | p <0.05 (ns after Bonferroni correction) | |
| Isoleucine | (Yang et al., 2013) | GC-TOFMS, ^1^H NMR | urine | ↑ | SCZ (N=51; DF ≥ 2 weeks, some FE) v HC (N=51) | - | p <0.04; q <0.045 | |
| Tryptophan | (Xuan et al., 2011) | GC-MS | serum | ↓ | DF SCZ (N=18 ; 10M; age = 38 ± 15); HC (N=18 ; 10M; age = 41 ± 19) | - | p = 0.0071 | |
|  | (Cao et al., 2018) | LC-MS | plasma | ↓ | SCZ (N = 208 (40DN&FE, 168 DF > 1 month); 135M; age = 37.77 ± 10.85)v HC (N=175; 54M; age = 39.44 ± 9.36) | + | P < 0.001 | |
| L-tryptophan | (Fukushima et al., 2014) | LC-MS | serum | ↑ | med SCZ (N=25; 11M; age = 28.2 ± 4.4) v HC (N=27; 12M; age = 26.5 ± 5.6) | + | p = 0.001 (also sig after Bonferroni correction) | |
| Valine | (Cai et al., 2012) | ^1^H NMR | urine | ↑ | FE DN SCZ (N=11; 6M; age = 27.6 ± 9.5) v HC (N=11; 6M; age = 27.6 ± 9.5) | - | p = 0.011 (ns after Bonferoni correction) | |
|  | (Yang et al., 2013) | GC-TOFMS, ^1^H NMR | urine | ↑ | SCZ (N=51; DF ≥ 2 weeks, some FE) v HC (N=51) | - | p <0.04; q <0.045 | |
|  | (Liu et al., 2014) | GC-MS | PBMCs | ↓ | SCZ (N=45; 18M; 19 FE DN, 26 med; age = 33.2 ± 12.9) v HC (N=50; 22M; age = 37.3 ± 8.7) | + | p <0.02 | |
| **Other organic acids and derivatives** | | | | | | | |  |
| 2-Oxoglutarate | (Yang et al., 2013) | GC-TOFMS, ^1^H NMR | serum | ↑ | SCZ (N=62; 25M; age = 36.9 ± 11.9; DF ≥ 2 weeks, some FE) v HC (N=62; 25M; age = 36.9 ± 9.3) | - | p <0.003; q <0.035 | |
| Cis-aconitic acid | (Yang et al., 2013) | GC-TOFMS, ^1^H NMR | urine | ↑ | SCZ (N=51; DF ≥ 2 weeks, some FE) v HC (N=51) | - | p = 0.0123, q = 0.033 | |
| 2,3-dihydroxybutanoic acid | (Yang et al., 2013) | GC-TOFMS, ^1^H NMR | urine | ↓ | SCZ (N=51; DF ≥ 2 weeks, some FE) v HC (N=51) | - | p <0.007, q <0.025 | |
| Citrate | (Xuan et al., 2011) | GC-MS | serum | ↓ | DF SCZ (N=18 ; 10M; age = 38 ± 15); HC (N=18 ; 10M; age = 41 ± 19) | - | p <0.04 | |
|  | (Cai et al., 2012) | ^1^H NMR | urine; plasma | ↓ | FE DN SCZ (N=11; 6M; age = 27.6 ± 9.5) v HC (N=11; 6M; age = 27.6 ± 9.5) | - | p <0.05 (ns after Bonferoni correction) | |
|  | (Yang et al., 2013) | GC-TOFMS, ^1^H NMR | serum | ↑ | SCZ (N=62; 25M; age = 36.9 ± 11.9; DF ≥ 2 weeks, some FE) v HC (N=62; 25M; age = 36.9 ± 9.3) | - | p <0.003; q <0.035 | |
|  | (Yang et al., 2013) | GC-TOFMS, ^1^H NMR | urine | ↓ | SCZ (N=41 SCZ; DF ≥ 2 weeks, some FE) v HC (N=41) | - | p = 0.00558 | |
|  | (Liu et al., 2015) | GC-MS | PBMCs | ↓ | Training set: FE DN SCZ (N=35; 14M; age = 32.5 ± 14.1) v HC (N=35; 18M; age = 36.5 ± 6.0); FE DN SCZ v DF MD (N=35; 17M; age = 36.4 ± 10.7)  Test set: SCZ (N=20; 9M; 6 med; age = 28.5 ± 2.1) v HC (N=20; 10M; age = 30.2 ± 1.7); FE SCZ v DF MD (N=20; 11M; age = 27.6 ± 2.1) | -, + | P <0.001 training set  p = 0.001 test set - | |
| Erythrose | (Xuan et al., 2011) | GC-MS | serum | ↑ | DF SCZ (N=18 ; 10M; age = 38 ± 15); HC (N=18 ; 10M; age = 41 ± 19) | - | p < 0.05 | |
| Fructose | (Liu et al., 2015) | GC-MS | PBMCs | ↑ | Training set: FE DN SCZ (N=35; 14M; age = 32.5 ± 14.1) v HC (N=35; 18M; age = 36.5 ± 6.0); FE DN SCZ v DF MD (N=35; 17M; age = 36.4 ± 10.7)  Test set: SCZ (N=20; 9M; 6 med; age = 28.5 ± 2.1) v HC (N=20; 10M; age = 30.2 ± 1.7); FE SCZ v DF MD (N=20; 11M; age = 27.6 ± 2.1) | -, + | P <0.001 training set  p = 0.003 test set - | |
| Fructose 6-phosphate | (Liu et al., 2015) | GC-MS | PBMCs | ↑ | Training set: FE DN SCZ (N=35; 14M; age = 32.5 ± 14.1) v HC (N=35; 18M; age = 36.5 ± 6.0); FE DN SCZ v DF MD (N=35; 17M; age = 36.4 ± 10.7)  Test set: SCZ (N=20; 9M; 6 med; age = 28.5 ± 2.1) v HC (N=20; 10M; age = 30.2 ± 1.7); FE SCZ v DF MD (N=20; 11M; age = 27.6 ± 2.1) | -, + | P <0.001 training and test sets | |
| Fumaric acid | (Liu et al., 2014) | GC-MS | PBMCs | ↓ | SCZ (N=45; 18M; 19 FE DN, 26 med; age = 33.2 ± 12.9) v HC (N=50; 22M; age = 37.3 ± 8.7) | + | p = 1.98 × 10−03 | |
| Galactose oxime | (Al Awam et al., 2015) | FTIR, GC-MS | serum | ↓ | med SCZ (N=26; 20M; age = 37.3 ± 12.4) v HC (N=26; 20M; age = 37.0 ± 10.7) | + | p <0.001 | |
| Glucose | (Xuan et al., 2011) | GC-MS | serum | ↑ | DF SCZ (N=18 ; 10M; age = 38 ± 15); HC (N=18 ; 10M; age = 41 ± 19) | - | p < 0.05 | |
|  | (Cai et al., 2012) | ^1^H NMR | plasma | ↓ | FE DN SCZ (N=11; 6M; age = 27.6 ± 9.5) v HC (N=11; 6M; age = 27.6 ± 9.5) | - | p = 0.045 (ns after Bonferoni correction) | |
|  | (Cai et al., 2012) | ^1^H NMR | Urine | ↑ |  |  | p = 0.0171 (ns after Bonferoni correction) | |
|  | (Yang et al., 2013) | GC-TOFMS, ^1^H NMR | urine | ↑ | SCZ (N=41 SCZ; DF ≥ 2 weeks, some FE) v HC (N=41) | - | p = 1.73 x 10-4 | |
|  | (Liu et al., 2015) | GC-MS | PBMCs | ↑ | Training set: FE DN SCZ (N=35; 14M; age = 32.5 ± 14.1) v HC (N=35; 18M; age = 36.5 ± 6.0); FE DN SCZ v DF MD (N=35; 17M; age = 36.4 ± 10.7)  Test set: SCZ (N=20; 9M; 6 med; age = 28.5 ± 2.1) v HC (N=20; 10M; age = 30.2 ± 1.7); FE SCZ v DF MD (N=20; 11M; age = 27.6 ± 2.1) | -, + | P <0.001 training set  p <0.001 test set | |
| Glucose 6-phosphate | (Liu et al., 2015) | GC-MS | PBMCs | ↑ | Training set: FE DN SCZ (N=35; 14M; age = 32.5 ± 14.1) v HC (N=35; 18M; age = 36.5 ± 6.0); FE DN SCZ v DF MD (N=35; 17M; age = 36.4 ± 10.7)  Test set: SCZ (N=20; 9M; 6 med; age = 28.5 ± 2.1) v HC (N=20; 10M; age = 30.2 ± 1.7); FE SCZ v DF MD (N=20; 11M; age = 27.6 ± 2.1) | -, + | P <0.001 training and test sets | |
| Gluconic acid | (Koike et al., 2014) | CE-TOFMS | plasma | ↑ | 1^st^ set: FE SCZ (N= 18; 13M; most med, 2 DN; 4 disorganised, 7 paranoid, 3 SFD, 2 delusional disorder, 2 PD NOS; age = 23.2 ± 5.4) v HC (N=14; 11M; age = 25.7 ± 6.1) | + | p = 0.022 | |
| Glucuronic acid | (Xuan et al., 2011) | GC-MS | serum | ↑ | DF SCZ (N=18 ; 10M; age = 38 ± 15); HC (N=18 ; 10M; age = 41 ± 19) | - | p < 0.05 | |
| Glyceraldehyde-3-phosphate | (Liu et al., 2015) | GC-MS | PBMCs | ↓ | Training set: FE DN SCZ (N=35; 14M; age = 32.5 ± 14.1) v HC (N=35; 18M; age = 36.5 ± 6.0); FE DN SCZ v DF MD (N=35; 17M; age = 36.4 ± 10.7)  Test set: SCZ (N=20; 9M; 6 med; age = 28.5 ± 2.1) v HC (N=20; 10M; age = 30.2 ± 1.7); FE SCZ v DF MD (N=20; 11M; age = 27.6 ± 2.1) | -, + | P <0.001 training set  p ≤0.006 test set | |
| Glycerate | (Yang et al., 2013) | GC-TOFMS, ^1^H NMR | serum | ↑ | SCZ (N=62; 25M; age = 36.9 ± 11.9; DF ≥ 2 weeks, some FE) v HC (N=62; 25M; age = 36.9 ± 9.3) | - | p <0.003; q <0.035 | |
| 1,3-Bisphosphoglycerate | (Xuan et al., 2011) | GC-MS | serum | ↓ | DF SCZ (N=18 ; 10M; age = 38 ± 15); HC (N=18 ; 10M; age = 41 ± 19) | - | p <0.04 | |
| Glycerate 3-phosphate | (Liu et al., 2015) | GC-MS | PBMCs | ↑ | Training set: FE DN SCZ (N=35; 14M; age = 32.5 ± 14.1) v HC (N=35; 18M; age = 36.5 ± 6.0); FE DN SCZ v DF MD (N=35; 17M; age = 36.4 ± 10.7)  Test set: SCZ (N=20; 9M; 6 med; age = 28.5 ± 2.1) v HC (N=20; 10M; age = 30.2 ± 1.7); FE SCZ v DF MD (N=20; 11M; age = 27.6 ± 2.1) | -, + | P <0.001 training set  p = 0.363 (ns) test set | |
| Glycerol | (Xuan et al., 2011) | GC-MS | serum | ↑ | DF SCZ (N=18 ; 10M; age = 38 ± 15); HC (N=18 ; 10M; age = 41 ± 19) | - | p < 0.05 | |
|  | (Liu et al., 2014) | GC-MS | PBMCs | ↑ | SCZ (N=45; 18M; 19 FE DN, 26 med; age = 33.2 ± 12.9) v HC (N=50; 22M; age = 37.3 ± 8.7) | + | 7.64 × 10−03 | |
| Inositol | (Liu et al., 2014) | GC-MS | PBMCs | ↓ | SCZ (N=45; 18M; 19 FE DN, 26 med; age = 33.2 ± 12.9) v HC (N=50; 22M; age = 37.3 ± 8.7) | + | p <0.035 | |
| Myo-inositol | (Xuan et al., 2011) | GC-MS | serum | ↑ | DF SCZ (N=18 ; 10M; age = 38 ± 15); HC (N=18 ; 10M; age = 41 ± 19) | - | p < 0.05 | |
|  | (Yang et al., 2013) | GC-TOFMS, ^1^H NMR | serum | ↑ | SCZ (N=62; 25M; age = 36.9 ± 11.9; DF ≥ 2 weeks, some FE) v HC (N=62; 25M; age = 36.9 ± 9.3) | - | p <0.003; q <0.035 | |
| Hydroxyacetic acid | (Yang et al., 2013) | GC-TOFMS, ^1^H NMR | urine | ↓ | SCZ (N=51; DF ≥ 2 weeks, some FE) v HC (N=51) | - | p <0.007, q <0.025 | |
| Lactobionic acid | (Xuan et al., 2011) | GC-MS | serum | ↑ | DF SCZ (N=18 ; 10M; age = 38 ± 15); HC (N=18 ; 10M; age = 41 ± 19) | - | p < 0.05 | |
| Lactatic acid | (Xuan et al., 2011) | GC-MS | serum | ↑ | DF SCZ (N=18 ; 10M; age = 38 ± 15); HC (N=18 ; 10M; age = 41 ± 19) | - | p < 0.05 | |
|  | (Cai et al., 2012) | ^1^H NMR | plasma | ↑ | FE DN SCZ (N=11; 6M; age = 27.6 ± 9.5) v HC (N=11; 6M; age = 27.6 ± 9.5) | - | p = 0.009 (ns after Bonferoni correction) | |
|  | (Yang et al., 2013) | GC-TOFMS, ^1^H NMR | serum | ↑ | SCZ (N=62; 25M; age = 36.9 ± 11.9; DF ≥ 2 weeks, some FE) v HC (N=62; 25M; age = 36.9 ± 9.3) | - | p <0.003; q <0.035 | |
|  | (Yang et al., 2013) | GC-TOFMS, ^1^H NMR | urine | ↓ | SCZ (N=41 SCZ; DF ≥ 2 weeks, some FE) v HC (N=41) | - | p = 2.09 x 10-8 | |
|  | (Liu et al., 2015) | GC-MS | PBMCs | ↓ | Training set: FE DN SCZ (N=35; 14M; age = 32.5 ± 14.1) v HC (N=35; 18M; age = 36.5 ± 6.0)  Test set: SCZ (N=20; 9M; 6 med; age = 28.5 ± 2.1) v HC (N=20; 10M; age = 30.2 ± 1.7) | -, + | p =ns training set  p = 0.002 test set | |
|  | Ours | CE-TOFMS/ LC-TOFMS | serum | ↑ | SCZ (N=20; 10M; 43.4 ± 8.8) v HC (N=20; 10M; age = 41.8 ± 12.2) | + | p = 7.6 × 10-7 | |
| D-lactatic acid | (Fukushima et al., 2014) | LC-MS | serum | ↑ | med SCZ (N=25; 11M; age = 28.2 ± 4.4) v HC (N=27; 12M; age = 26.5 ± 5.6) | + | p = 2.43 x 10-5 (also sig after Bonferroni correction) | |
| Maltose | (Liu et al., 2014) | GC-MS | PBMCs | ↓ | SCZ (N=45; 18M; 19 FE DN, 26 med; age = 33.2 ± 12.9) v HC (N=50; 22M; age = 37.3 ± 8.7) | + | p <0.035 | |
| Methyl phosphate | (Liu et al., 2014) | GC-MS | PBMCs | ↓ | SCZ (N=45; 18M; 19 FE DN, 26 med; age = 33.2 ± 12.9) v HC (N=50; 22M; age = 37.3 ± 8.7) | + | p <0.01 | |
| Proline | (Parksepp et al., 2020) | FIA-MS, LC-MS | serum | ↓ | DN FEP (N=52; 31M; age = 27.0 ± 6.1) v HC (N=37; 16M; age = 24.9 ± 5.3) | - | p = 0.0002 | |
| Pyruvate | (Yang et al., 2013) | GC-TOFMS, ^1^H NMR | serum | ↑ | SCZ (N=62; 25M; age = 36.9 ± 11.9; DF ≥ 2 weeks, some FE) v HC (N=62; 25M; age = 36.9 ± 9.3) | - | p <0.003; q <0.035 | |
|  | (Liu et al., 2015) | GC-MS | PBMCs | ↑ | Training set: FE DN SCZ (N=35; 14M; age = 32.5 ± 14.1) v HC (N=35; 18M; age = 36.5 ± 6.0)  Test set: SCZ (N=20; 9M; 6 med; age = 28.5 ± 2.1) v HC (N=20; 10M; age = 30.2 ± 1.7) | -, + | P = ns training set  p = 0.013 test set | |
| Ribose 5-phosphate | (Liu et al., 2015) | GC-MS | PBMCs | ↑ | Training set: FE DN SCZ (N=35; 14M; age = 32.5 ± 14.1) v HC (N=35; 18M; age = 36.5 ± 6.0)  Test set: SCZ (N=20; 9M; 6 med; age = 28.5 ± 2.1) v HC (N=20; 10M; age = 30.2 ± 1.7) | -, + | P <0.001 training set  p = ns test set | |
| Sorbitol | (Liu et al., 2015) | GC-MS | PBMCs | ↓ | SCZ (N=45; 18M; 19 FE DN, 26 med; age = 33.2 ± 12.9) v HC (N=50; 22M; age = 37.3 ± 8.7) | + | p <10^−5^ | |
| Succinic acid | (Liu et al., 2015) | GC-MS | PBMCs | ↑ | Training set: FE DN SCZ (N=35; 14M; age = 32.5 ± 14.1) v HC (N=35; 18M; age = 36.5 ± 6.0)  Test set: SCZ (N=20; 9M; 6 med; age = 28.5 ± 2.1) v HC (N=20; 10M; age = 30.2 ± 1.7) | -, + | P <0.001 training set & test set | |
| **Clusters** | | | | | | | |  |
| MC3 (Branched chain amino acids and other amino acids; e.g. Isoleucine, phenylalanine, tyrosine, ornithine, serine, methionine, threonine) | (Orešič et al., 2011) | GC-TOFMS | serum | ↑ | SCZ (N=45; 19M; 34 med; age = 53.7 ± 12.9) v HC (N=45; 19M; age = 53.7 ± 12.9) | + | p = 0.045 | |
| MC5 (Amino acids, organic acids e.g. proline, glutamic acid, α-ketoglutaric acid, pyruvic acid, alanine, lactic acid, α-hydroxybutyrate) | (Orešič et al., 2011) | GC-TOFMS | serum | ↑ | SCZ (N=45; 19M; 34 med; age = 53.7 ± 12.9) v HC (N=45; 19M; age = 53.7 ± 12.9) | + | p = 0.020 | |

α-KG = α-ketoglutarate ; GC = gas chromatography; TOF-MS = time-of-flight mass spectrometry; ^1^H NMR = proton nuclear magnetic resonance spectroscopy; UPLC-MS/MS = ultra-performance liquid chromatography tandem mass-spectrometry; HPLC = high-performance liquid chromatography; LC = liquid chromatography; FIA-MS = flow injection analysis MS; CE = capillary electrophoresis; PBMCs = peripheral blood mononuclear cells; SCZ = schizophrenia subjects; SFD = schizophreniform disorder; SZA = schizoaffective disorder; HC = healthy control subjects; AP = antipsychotic medication; DN = drug naïve ; ns = not significant; FE = first episode; MD = major depressive disorder; ASD = autism spectrum disorder; PD NOS = psychotic disorder not otherwise specified

**Table s6: Other metabolites found at abnormal levels in subjects with schizophrenia**

| **Metabolite** | **Reference** | **Platform** | **Biofluid** | **Difference (SCZ v control)** | **Subjects** | **Medication** | **P-value** |
| --- | --- | --- | --- | --- | --- | --- | --- |
| 3-Indolebutyrate fragments | (Cai et al., 2012) | ^1^H NMR | plasma | ↑ | FE DN SCZ (N=11; 6M; age = 27.6 ± 9.5) v HC (N=11; 6M; age = 27.6 ± 9.5) | - | p = 0.045 (ns after Bonferoni correction) |
| 5-HT | (Fukushima et al., 2014) | LC-MS | serum | ↓ | med SCZ (N=25; 11M; age = 28.2 ± 4.4) v HC (N=27; 12M; age = 26.5 ± 5.6) | + | p = 0.013 (ns after Bonferroni correction) |
| 6-deoxy-mannofuranose | (Al Awam et al., 2015) | FTIR, GC-MS | serum | ↓ | med SCZ (N=26; 20M; age = 37.3 ± 12.4) v HC (N=26; 20M; age = 37.0 ± 10.7) | + | p <0.001 |
| α-KG | (Xuan et al., 2011) | GC-MS | serum | ↓ | DF SCZ (N=18 ; 10M; age = 38 ± 15); HC (N=18 ; 10M; age = 41 ± 19) | - | p <0.04 |
|  | (Cai et al., 2012) | ^1^H NMR | urine; plasma | ↓ | FE DN SCZ (N=11; 6M; age = 27.6 ± 9.5) v HC (N=11; 6M; age = 27.6 ± 9.5) | - | p <0.05 (ns after Bonferoni correction) |
| Acetoacetate | (Cai et al., 2012) | ^1^H NMR | urine; plasma | ↓ | FE DN SCZ (N=11; 6M; age = 27.6 ± 9.5) v HC (N=11; 6M; age = 27.6 ± 9.5) | - | p <0.05 (ns after Bonferoni correction) |
| Dihydroxyacetone phosphate | (Liu et al., 2015) | GC-MS | PBMCs | ↓ | Training set: FE DN SCZ (N=35; 14M; age = 32.5 ± 14.1) v HC (N=35; 18M; age = 36.5 ± 6.0)  Test set: SCZ (N=20; 9M; 6 med; age = 28.5 ± 2.1) v HC (N=20; 10M; age = 30.2 ± 1.7) | -, + | P <0.001 training set  p ≤0.006 test set |
| Adrenaline; noradenaline | (Fryar-Williams and Strobel, 2015) | MS | urine | ↑ | SCZ (N=67; 37M; SCZ & SZA; med and DF; age = 40.5 ± 1.3) v HC (N=67; 33M; age = 45.7 ±1.4) | + | p <0.0001 |
| Allantoin | (Xuan et al., 2011) | GC-MS | serum | ↑ | DF SCZ (N=18 ; 10M; age = 38 ± 15); HC (N=18 ; 10M; age = 41 ± 19) | - | p = 0.0298 |
| Benzoic acid; hydroxylamine | (Liu et al., 2014) | GC-MS | PBMCs | ↑ | SCZ (N=45; 18M; 19 FE DN, 26 med; age = 33.2 ± 12.9) v HC (N=50; 22M; age = 37.3 ± 8.7) | + | p <0.03 |
| Benzoic acid | (Koike et al., 2014) | CE-TOFMS | plasma | ↓ | 1^st^ set: FE SCZ (N= 18; 13M; most med, 2 DN; 4 disorganised, 7 paranoid, 3 SFD, 2 delusional disorder, 2 PD NOS; age = 23.2 ± 5.4) v HC (N=14; 11M; age = 25.7 ± 6.1)  2^nd^ set (only benzoic acid detected): FE SCZ (N=12; 4 disorganised, 3 paranoid, 3 SFD, 2 PD NOS; 2 DN; age = 24.6 ± 7.1) v HC (N=24 HC; 10M; age = 26.1 ± 2.6) | + | p = 0.037 (1st set)  p = 0.039 (2nd set) |
| Catechol | (Yang et al., 2013) | GC-TOFMS, ^1^H NMR | urine | ↓ | SCZ (N=51; DF ≥ 2 weeks, some FE) v HC (N=51) | - | p = 5.82 x 10^-4^, q = 0.00748 |
| Free copper to zinc ratio | (Fryar-Williams and Strobel, 2015) | MS | serum/ RBCs | ↑ | SCZ (N=67; 37M; SCZ & SZA; med and DF; age = 40.5 ± 1.3) v HC (N=67; 33M; age = 45.7 ±1.4) | + | p = 0.0104 |
| Cyclohexylamine | (Koike et al., 2014) | CE-TOFMS | plasma | ↓ | 1^st^ set: FE SCZ (N= 18; 13M; most med, 2 DN; 4 disorganised, 7 paranoid, 3 SFD, 2 delusional disorder, 2 PD NOS; age = 23.2 ± 5.4) v HC (N=14; 11M; age = 25.7 ± 6.1)  2^nd^ set (only benzoic acid detected): FE SCZ (N=12; 4 disorganised, 3 paranoid, 3 SFD, 2 PD NOS; 2 DN; age = 24.6 ± 7.1) v HC (N=24 HC; 10M; age = 26.1 ± 2.6) | + | p = 0.0018 (1st set)  not detected in 2nd set |
| Dopamine | (Liu et al., 2014) | GC-MS | PBMCs | ↓ | SCZ (N=45; 18M; 19 FE DN, 26 med; age = 33.2 ± 12.9) v HC (N=50; 22M; age = 37.3 ± 8.7) | + | 4.89 × 10^−02^ |
|  | (Fryar-Williams and Strobel, 2015) | MS | urine | ↑ | SCZ (N=67; 37M; SCZ & SZA; med and DF; age = 40.5 ± 1.3) v HC (N=67; 33M; age = 45.7 ±1.4) | + | p <0.0001 |
| Folate | (Fryar-Williams and Strobel, 2015) | MS | RBCs | ↓ | SCZ (N=67; 37M; SCZ & SZA; med and DF; age = 40.5 ± 1.3) v HC (N=67; 33M; age = 45.7 ± 1.4) | + | p = 0.0005 |
| Hippurate | (Cai et al., 2012) | ^1^H NMR | urine | ↓ | FE DN SCZ (N=11; 6M; age = 27.6 ± 9.5) v HC (N=11; 6M; age = 27.6 ± 9.5) | - | p <0.0001 (UPLC-MS/MS)  p = 0.001 (^1^H NMR) (still sig. after Bonferoni correction) |
| Imidazolelactic acid | (Koike et al., 2014) | CE-TOFMS | plasma | ↓ | 1^st^ set: FE SCZ (N= 18; 13M; most med, 2 DN; 4 disorganised, 7 paranoid, 3 SFD, 2 delusional disorder, 2 PD NOS; age = 23.2 ± 5.4) v HC (N=14; 11M; age = 25.7 ± 6.1)  2^nd^ set (only benzoic acid detected): FE SCZ (N=12; 4 disorganised, 3 paranoid, 3 SFD, 2 PD NOS; 2 DN; age = 24.6 ± 7.1) v HC (N=24 HC; 10M; age = 26.1 ± 2.6) | + | p = 0.037 (1st set)  not detected in 2nd set |
| Kynurenine | (Kegel et al., 2014) | LC-MS | CSF | ↑ | SCZ (N=21; age = 37.5 ± 7.5 ) v HC (N=26; age = 24.9 ± 5.8) | + | p = 0.001 |
|  | (Cao et al., 2018) | LC-MS | plasma | ↓ | SCZ (N = 208 (40DN&FE, 168 DF > 1 month); 135M; age = 37.77 ± 10.85)v HC (N=175; 54M; age = 39.44 ± 9.36) | + | p < 0.001 |
| L-kynurenine | (Fukushima et al., 2014) | LC-MS | serum | ↑ | med SCZ (N=25; 11M; age = 28.2 ± 4.4) v HC (N=27; 12M; age = 26.5 ± 5.6) | + | p = 0.0057 (ns after Bonferroni correction) |
| ﻿3-hydroxykynurenine | (De Picker et al., 2020) | LC-MS | plasma | ↓ | SCZ (N=49; 42M; age = 32.4 ± 7.5, DN) v HC (N=52; 39M; age = 28.5 ± 7.0) | - | p <0.001 |
| KYNA | (Kegel et al., 2014) | LC-MS | CSF | ↑ | SCZ (N=21; age = 37.5 ± 7.5 ) v HC (N=26; age = 24.9 ± 5.8) | + | p = 0.012 |
|  | (Wurfel et al., 2017) | LC-MS | serum | ↓ | SCZ-A (N=40; 24M; age = 39.0 ± 13.0) v HC (N=92; 33M; age = 32.3 ± 10.4) | + | p <0.001 |
|  | (Huang et al., 2020) | LC-MS | plasma | ↑ | SCZ (N=30; 15M; age = 27.63 ± 7.23, 21 DN) v HC (N=34; 13M; age = 29.59 ± 8.36) | + | p = 0.009 |
|  | (De Picker et al., 2020) | LC-MS | plasma | ↓ | SCZ (N=49; 42M; age = 32.4 ± 7.5, DN) v HC (N=52; 39M; age = 28.5 ± 7.0) | - | p <0.001 |
| KYNA/tryptophan | (Huang et al., 2020) | LC-MS | plasma | ↑ | SCZ (N=30; 15M; age = 27.63 ± 7.23, 21 DN) v HC (N=34; 13M; age = 29.59 ± 8.36) | + | p = 0.004 |
| KYNA/Kyn | (De Picker et al., 2020) | LC-MS | plasma | ↓ | SCZ (N=49; 42M; age = 32.4 ± 7.5, DN) v HC (N=52; 39M; age = 28.5 ± 7.0) | - | p <0.001 |
| KYNA/quinolinic acid | (Wurfel et al., 2017) | LC-MS | serum | ↓ | SCZ-A (N=40; 24M; age = 39.0 ± 13.0) v HC (N=92; 33M; age = 32.3 ± 10.4) | + | p = 0.001 |
| KYNA/ 3HK | (Wurfel et al., 2017) | LC-MS | serum | ↓ | SCZ-A (N=40; 24M; age = 39.0 ± 13.0) v HC (N=92; 33M; age = 32.3 ± 10.4) | + | P = 0.004 |
| Quinolinic acid | (De Picker et al., 2020) | LC-MS | plasma | ↓ | SCZ (N=49; 42M; age = 32.4 ± 7.5, DN) v HC (N=52; 39M; age = 28.5 ± 7.0) | - | p <0.001 |
| NO_x_ | (De Picker et al., 2020) | LC-MS | plasma | ↓ | SCZ (N=49; 42M; age = 32.4 ± 7.5, DN) v HC (N=52; 39M; age = 28.5 ± 7.0) | - | p <0.001 |
| Tocopherol-α | (Liu et al., 2014) | GC-MS | PBMCs | ↓ | SCZ (N=45; 18M; 19 FE DN, 26 med; age = 33.2 ± 12.9) v HC (N=50; 22M; age = 37.3 ± 8.7) | + | p = 4.39 × 10^−4^ |
| Tocopherol-γ | (Liu et al., 2014) | GC-MS | PBMCs | ↓ | SCZ (N=45; 18M; 19 FE DN, 26 med; age = 33.2 ± 12.9) v HC (N=50; 22M; age = 37.3 ± 8.7) | + | p = 4.75 × 10^−3^ |
| Tocopherol- γ | (Xuan et al., 2011) | GC-MS | serum | ↓ | DF SCZ (N=18 ; 10M; age = 38 ± 15); HC (N=18 ; 10M; age = 41 ± 19) | - | p = 0.0248 |
| Urea | Ours | CE-TOFMS/ LC-TOFMS | serum | ↓ | SCZ (N=20; 10M; 43.4 ± 8.8) v HC (N=20; 10M; age = 41.8 ± 12.2) | + | p = 2.9 × 10^-5^ |
| Uric acid | (Xuan et al., 2011) | GC-MS | serum | ↓ | DF SCZ (N=18 ; 10M; age = 38 ± 15); HC (N=18 ; 10M; age = 41 ± 19) | - | p = 0.0247 |
|  | (Cai et al., 2012) | ^1^H NMR | plasma | ↓ | FE DN SCZ (N=11; 6M; age = 27.6 ± 9.5) v HC (N=11; 6M; age = 27.6 ± 9.5) | - | p = 0.023 (ns after Bonferoni correction) |
|  | (Cai et al., 2012) | ^1^H NMR | urine | ↑ | FE DN SCZ (N=11; 6M; age = 27.6 ± 9.5) v HC (N=11; 6M; age = 27.6 ± 9.5) | - | p = 0.033 (ns after Bonferoni correction) |
| Vitamin B6 | (Fryar-Williams and Strobel, 2015) | MS | whole blood | ↓ | SCZ (N=67; 37M; SCZ & SZA; med and DF; age = 40.5 ± 1.3) v HC (N=67; 33M; age = 45.7 ± 1.4) | + | p = 0.0009 |
| Vitamin D | (Fryar-Williams and Strobel, 2015) | MS | blood | ↓ | SCZ (N=67; 37M; SCZ & SZA; med and DF; age = 40.5 ± 1.3) v HC (N=67; 33M; age = 45.7 ± 1.4) | + | p = 0.0026 |
| TMAO | (Cai et al., 2012) | ^1^H NMR | Urine | ↓ | FE DN SCZ (N=11; 6M; age = 27.6 ± 9.5) v HC (N=11; 6M; age = 27.6 ± 9.5) | - | p = 0.028 (ns after Bonferoni correction) |

KYNA = kynurenic acid; 5-HT = 5-hydroxy tryptamine (serotonin); NA5HT = N-acetylserotonin; 5-HTP = 5-hydroxy tryptophan; NO_x_ = nitric oxide and metabolites; TMAO = trimethylamine-N-oxide; MS = mass spectrometry; GC = gas chromatography; CE-TOFMS = capillary electrophoresis time-of-flight MS; ^1^H NMR = proton nuclear magnetic resonance spectroscopy; RBCs = red blood cells; PBMCs = peripheral blood mononuclear cells; CSF = cerebrospinal fluid; SCZ = schizophrenia subjects; SZA = schizoaffective disorder; HC = healthy control subjects; AP = antipsychotic medication; DN = drug naïve; FE = first episode; ns = not significant

**Reference**

Al Awam, K., Haußleiter, I.S., Dudley, E., Donev, R., Brüne, M., Juckel, G., Thome, J., 2015. Multiplatform metabolome and proteome profiling identifies serum metabolite and protein signatures as prospective biomarkers for schizophrenia. J. Neural Transm. 122, 111–122. https://doi.org/10.1007/s00702-014-1224-0

Ballesteros, A., Summerfelt, A., Du, X., Jiang, P., Chiappelli, J., Tagamets, M., O’Donnell, P., Kochunov, P., Hong, L.E., 2013. Electrophysiological intermediate biomarkers for oxidative stress in schizophrenia. Clin. Neurophysiol. 124, 2209–2215. https://doi.org/10.1016/j.clinph.2013.05.021

Bicikova, M., Hill, M., Ripova, D., Mohr, P., Hampl, R., 2013. Determination of steroid metabolome as a possible tool for laboratory diagnosis of schizophrenia. J. Steroid Biochem. Mol. Biol. 133, 77–83. https://doi.org/10.1016/j.jsbmb.2012.08.009

Cai, H.L., Li, H. De, Yan, X.Z., Sun, B., Zhang, Q., Yan, M., Zhang, W.Y., Jiang, P., Zhu, R.H., Liu, Y.P., Fang, P.F., Xu, P., Yuan, H.Y., Zhang, X.H., Hu, L., Yang, W., Ye, H. Sen, 2012. Metabolomic analysis of biochemical changes in the plasma and urine of first-episode neuroleptic-naïve schizophrenia patients after treatment with risperidone. J. Proteome Res. 11, 4338–4350. https://doi.org/10.1021/pr300459d

Cao, B., Wang, D., Brietzke, E., McIntyre, R.S., Pan, Z., Cha, D., Rosenblat, J.D., Zuckerman, H., Liu, Y., Xie, Q., Wang, J., 2018. Characterizing amino-acid biosignatures amongst individuals with schizophrenia: a case–control study. Amino Acids 50, 1013–1023. https://doi.org/10.1007/s00726-018-2579-6

Cao, B., Wang, D., Pan, Z., Brietzke, E., McIntyre, R.S., Musial, N., Mansur, R.B., Subramanieapillai, M., Zeng, J., Huang, N., Wang, J., 2019. Characterizing acyl-carnitine biosignatures for schizophrenia: a longitudinal pre- and post-treatment study. Transl. Psychiatry 9. https://doi.org/10.1038/s41398-018-0353-x

De Picker, L., Fransen, E., Coppens, V., Timmers, M., de Boer, P., Oberacher, H., Fuchs, D., Verkerk, R., Sabbe, B., Morrens, M., 2020. Immune and Neuroendocrine Trait and State Markers in Psychotic Illness: Decreased Kynurenines Marking Psychotic Exacerbations. Front. Immunol. 10, 1–12. https://doi.org/10.3389/fimmu.2019.02971

Fryar-Williams, S., Strobel, J.E., 2015. Biomarkers of a five-domain translational substrate for schizophrenia and schizoaffective psychosis. Biomark. Res. 3, 1–21. https://doi.org/10.1186/s40364-015-0028-1

Fukushima, T., Iizuka, H., Yokota, A., Suzuki, T., Ohno, C., Kono, Y., Nishikiori, M., Seki, A., Ichiba, H., Watanabe, Y., Hongo, S., Utsunomiya, M., Nakatani, M., Sadamoto, K., Yoshio, T., 2014. Quantitative analyses of schizophrenia-associated metabolites in serum: Serum D-lactate levels are negatively correlated with gamma-glutamylcysteine in medicated schizophrenia patients. PLoS One 9. https://doi.org/10.1371/journal.pone.0101652

He, Y., Yu, Z., Giegling, I., Xie, L., Hartmann, A.M., Prehn, C., Adamski, J., Kahn, R., Li, Y., Illig, T., Wang-Sattler, R., Rujescu, D., 2012. Schizophrenia shows a unique metabolomics signature in plasma. Transl. Psychiatry 2. https://doi.org/10.1038/tp.2012.76

Huang, N., Cao, B., Brietzke, E., Park, C., Cha, D., Pan, Z., Zhu, J., Liu, Y., Xie, Q., Zeng, J., McIntyre, R.S., Wang, J., Yan, L., 2019. A pilot case-control study on the association between N-acetyl derivatives in serum and first-episode schizophrenia. Psychiatry Res. 272, 36–41. https://doi.org/10.1016/j.psychres.2018.11.064

Huang, X., Ding, W., Wu, F., Zhou, S., Deng, S., Ning, Y., 2020. Increased plasma kynurenic acid levels are associated with impaired attention/vigilance and social cognition in patients with schizophrenia. Neuropsychiatr. Dis. Treat. 16, 263–271. https://doi.org/10.2147/NDT.S239763

Kageyama, Y., Kasahara, T., Morishita, H., Mataga, N., Deguchi, Y., Tani, M., Kuroda, K., Hattori, K., Yoshida, S., Inoue, K., Kato, T., 2017. Search for plasma biomarkers in drug-free patients with bipolar disorder and schizophrenia using metabolome analysis. Psychiatry Clin. Neurosci. 71, 115–123. https://doi.org/10.1111/pcn.12461

Kegel, M.E., Bhat, M., Skogh, E., Samuelsson, M., Lundberg, K., Dahl, M.L., Sellgren, C., Schwieler, L., Engberg, G., Schuppe-Koistinen, I., Erhardt, S., 2014. Imbalanced Kynurenine Pathway in Schizophrenia. Int. J. Tryptophan Res. 7, 15–22. https://doi.org/10.4137/IJTR.S16800

Koido, K., Innos, J., Haring, L., Zilmer, M., Ottas, A., Vasar, E., 2016. Taurine and epidermal growth factor belong to the signature of first-episode psychosis. Front. Neurosci. 10. https://doi.org/10.3389/fnins.2016.00331

Koike, S., Bundo, M., Iwamoto, K., Suga, M., Kuwabara, H., Ohashi, Y., Shinoda, K., Takano, Y., Iwashiro, N., Satomura, Y., Nagai, T., Natsubori, T., Tada, M., Yamasue, H., Kasai, K., 2014. A snapshot of plasma metabolites in first-episode schizophrenia: A capillary electrophoresis time-of-flight mass spectrometry study. Transl. Psychiatry 4, 1–8. https://doi.org/10.1038/tp.2014.19

Kriisa, K., Leppik, L., Balõtšev, R., Ottas, A., Soomets, U., Koido, K., Volke, V., Innos, J., Haring, L., Vasar, E., Zilmer, M., 2017. Profiling of acylcarnitines in first episode psychosis before and after antipsychotic treatment. J. Proteome Res. 16, 3558–3566. https://doi.org/10.1021/acs.jproteome.7b00279

Leppik, L., Parksepp, M., Janno, S., Koido, K., Haring, L., Vasar, E., Zilmer, M., 2020. Profiling of lipidomics before and after antipsychotic treatment in first-episode psychosis. Eur. Arch. Psychiatry Clin. Neurosci. 270, 59–70. https://doi.org/10.1007/s00406-018-0971-6

Liu, M.-L., Zheng, P., Liu, Z., Xu, Y., Mu, J., Guo, J., Huang, T., Meng, H.-Q., Xie, P., 2014. GC-MS based metabolomics identification of possible novel biomarkers for schizophrenia in peripheral blood mononuclear cells. Mol. BioSyst. 10, 2398–2406. https://doi.org/10.1039/C4MB00157E

Liu, M.L., Zhang, X.T., Du, X.Y., Fang, Z., Liu, Z., Xu, Y., Zheng, P., Xu, X.J., Cheng, P.F., Huang, T., Bai, S.J., Zhao, L.B., Qi, Z.G., Shao, W.H., Xie, P., 2015. Severe disturbance of glucose metabolism in peripheral blood mononuclear cells of schizophrenia patients: A targeted metabolomic study. J. Transl. Med. 13, 1–9. https://doi.org/10.1186/s12967-015-0540-y

Orešič, M., Seppänen-Laakso, T., Sun, D., Tang, J., Therman, S., Viehman, R., Mustonen, U., van Erp, T.G., Hyötyläinen, T., Thompson, P., Toga, A.W., Huttunen, M.O., Suvisaari, J., Kaprio, J., Lönnqvist, J., Cannon, T.D., 2012. Phospholipids and insulin resistance in psychosis: A lipidomics study of twin pairs discordant for schizophrenia. Genome Med. 4. https://doi.org/10.1186/gm300

Orešič, M., Tang, J., Seppänen-Laakso, T., Mattila, I., Saarni, S.E., Saarni, S.I., Lönnqvist, J., Sysi-Aho, M., Hyötyläinen, T., Perälä, J., Suvisaari, J., 2011. Metabolome in schizophrenia and other psychotic disorders: A general population-based study. Genome Med. 3. https://doi.org/10.1186/gm233

Parksepp, M., Leppik, L., Koch, K., Uppin, K., Kangro, R., Haring, L., 2020. Metabolomics approach revealed robust changes in amino acid and biogenic amine signatures in patients with schizophrenia in the early course of the disease. Sci. Rep. 1–11. https://doi.org/10.1038/s41598-020-71014-w

Schwarz, E., Prabakaran, S., Whitfield, P., Major, H., Leweke, F.M., Koethe, D., McKenna, P., Bahn, S., 2008. High throughput lipidomic profiling of schizophrenia and bipolar disorder brain tissue reveals alterations of free fatty acids, phosphatidylcholines, and ceramides. J. Proteome Res. 7, 4266–4277. https://doi.org/10.1021/pr800188y

Tessier, C., Sweers, K., Frajerman, A., Bergaoui, H., Ferreri, F., Delva, C., Lapidus, N., Lamaziere, A., Roiser, J.P., De Hert, M., Nuss, P., 2016. Membrane lipidomics in schizophrenia patients: A correlational study with clinical and cognitive manifestations. Transl. Psychiatry 6, e906-8. https://doi.org/10.1038/tp.2016.142

Tsang, T.M., Huang, J.T.J., Holmes, E., Bahn, S., 2006. Metabolic profiling of plasma from discordant schizophrenia twins: Correlation between lipid signals and global functioning in female schizophrenia patients. J. Proteome Res. 5, 756–760. https://doi.org/10.1021/pr0503782

Wang, D., Sun, X., Yan, J., Ren, B., Cao, B., Lu, Q., Liu, Y., Zeng, J., Huang, N., Xie, Q., Gu, H., Wang, J., 2018. Alterations of eicosanoids and related mediators in patients with schizophrenia. J. Psychiatr. Res. 102, 168–178. https://doi.org/10.1016/j.jpsychires.2018.04.002

Wurfel, B.E., Drevets, W.C., Bliss, S.A., McMillin, J.R., Suzuki, H., Ford, B.N., Morris, H.M., Teague, T.K., Dantzer, R., Savitz, J.B., 2017. Serum kynurenic acid is reduced in affective psychosis. Transl. Psychiatry 7. https://doi.org/10.1038/tp.2017.88

Xuan, J., Pan, G., Qiu, Y., Yang, L., Su, M., Liu, Y., Chen, J., Feng, G., Fang, Y., Jia, W., Xing, Q., He, L., 2011. Metabolomic profiling to identify potential serum biomarkers for schizophrenia and risperidone action. J. Proteome Res. 10, 5433–5443. https://doi.org/10.1021/pr2006796

Yan, L., Zhou, J., Wang, D., Si, D., Liu, Y., Zhong, L., Yin, Y., 2018. Unbiased lipidomic profiling reveals metabolomic changes during the onset and antipsychotics treatment of schizophrenia disease. Metabolomics 14, 1–13. https://doi.org/10.1007/s11306-018-1375-3

Yang, J., Chen, T., Sun, L., Zhao, Z., Qi, X., Zhou, K., Cao, Y., Wang, X., Qiu, Y., Su, M., Zhao, A., Wang, P., Yang, P., Wu, J., Feng, G., He, L., Jia, W., Wan, C., 2013. Potential metabolite markers of schizophrenia. Mol. Psychiatry 18, 67–78. https://doi.org/10.1038/mp.2011.131

Yang, X., Sun, L., Zhao, A., Hu, X., Qing, Y., Jiang, J., Yang, C., Xu, T., Wang, P., Liu, J., Zhang, J., He, L., Jia, W., Wan, C., 2017. Serum fatty acid patterns in patients with schizophrenia: a targeted metabonomics study. Transl. Psychiatry 7, e1176. https://doi.org/10.1038/tp.2017.152

Yoshikawa, A., Nishimura, F., Inai, A., Eriguchi, Y., Nishioka, M., Takaya, A., Tochigi, M., Kawamura, Y., Umekage, T., Kato, K., Sasaki, T., Ohashi, Y., Iwamoto, K., Kasai, K., Kakiuchi, C., 2018. Mutations of the glycine cleavage system genes possibly affect the negative symptoms of schizophrenia through metabolomic profile changes. Psychiatry Clin. Neurosci. 72, 168–179. https://doi.org/10.1111/pcn.12628
